# A general computational approach to predicting synergistic transcriptional cores that determine cell subpopulation identities

**DOI:** 10.1093/nar/gkz147

**Published:** 2019-03-01

**Authors:** Satoshi Okawa, Antonio del Sol

**Affiliations:** 1 Integrated BioBank of Luxembourg, Dudelange L-3555, Luxembourg; 2 Luxembourg Centre for Systems Biomedicine (LCSB), University of Luxembourg, 6, avenue du Swing, L-4367 Belvaux, Luxembourg; 3 CIC bioGUNE, Bizkaia Technology Park, 801 building, Derio 48160, Spain; 4 IKERBASQUE, Basque Foundation for Science, Bilbao 48013, Spain; 5 Moscow Institute of Physics and Technology, Dolgoprudny 141701, Russia

## Abstract

Advances in single-cell RNA-sequencing techniques reveal the existence of distinct cell subpopulations. Identification of transcription factors (TFs) that define the identity of these subpopulations poses a challenge. Here, we postulate that identity depends on background subpopulations, and is determined by a synergistic core combination of TFs mainly uniquely expressed in each subpopulation, but also TFs more broadly expressed across background subpopulations. Building on this view, we develop a new computational method for determining such synergistic identity cores of subpopulations within a given cell population. Our method utilizes an information-theoretic measure for quantifying transcriptional synergy, and implements a novel algorithm for searching for optimal synergistic cores. It requires only single-cell RNA-seq data as input, and does not rely on any prior knowledge of candidate genes or gene regulatory networks. Hence, it can be directly applied to any cellular systems, including those containing novel subpopulations. The method is capable of recapitulating known experimentally validated identity TFs in eight published single-cell RNA-seq datasets. Furthermore, some of these identity TFs are known to trigger cell conversions between subpopulations. Thus, this methodology can help design strategies for cell conversion within a cell population, guiding experimentalists in the field of stem cell research and regenerative medicine.

## INTRODUCTION

Advances in high-throughput single-cell RNA-sequencing (RNA-seq) technologies make it possible to profile RNA expression in thousands of individual cells and provide unprecedented resolution with which biological systems can be investigated. In particular, single-cell RNA-seq data for populations of cells reveal the existence, and enable the characterization of, distinct subpopulations of cells. In this way, single-cell RNA-seq data provide a more fine-grained, detailed information on populations of cells compared to the bulk transcriptomics data available for the same populations. Moreover, it has been reported that the expression of only a handful of specific TFs is enough to maintain the identity of a cell subpopulation (1). Knowledge of these TFs would not only provide characterization of subpopulation identities, but also enable the design of novel strategies for cell conversion between given subpopulations, including rare and even unknown ones (2).

Existing methods presented in the literature for the identification of TFs for conversion of cells from one type to another (3–5) depend essentially on bulk gene expression profiles of a pre-selected, fixed set of cell- or tissue types. Therefore, these methods are restricted to the types from this set alone and they cannot be applied to new subpopulations of cells revealed by single-cell RNA-seq measurements. Moreover, these methods consider individual TFs, rather than interconnected combinations of TFs that together define cell identity. However, considering such interconnectivity between TFs is important for the proper identification of core identity TFs according to recent reports as discussed below.

**Table 1.** Examples of synergistic identity cores obtained by our method divided into specific TF part and non-specific TF part

Data set	Cell subpopulation	Synergistic identity core (specific TFs)	Synergistic identity core (non-specific TFs)
Grün *et al.* 2015	Intestinal organoid enteroendocrine cell	POU6F1 MSX1 **NEUROD1 RFX6** HOXB6	ZMIZ2 JUND **LMX1A**
Zeisel *et al.* 2015	Cortical oligodendrocyte	**NKX6**-**2 MYRF SOX10 OLIG2**	**SP1** VEZF1 **NFIB**
Gokce *et al.* 2016	Striatal microglia	**MAFB** HHEX **ATF3** FLI1 ETS1	**JUN EGR1** FOXN2
Gokce *et al.* 2016	Striatal astrocyte	SOX21 **SOX9** SOX5 ID2 **SOX6**	TCF4 **NFIB** SMARCC2
Gokce *et al.* 2016	Striatal neuron	**MYT1L BCL11B BCL11A** TRERF1 NPAS2	TSC22D1 **MEIS2** PEG3
Gokce *et al.* 2016	Striatal oligodendrocyte	**SOX10 PROX1 OLIG2** LITAF **ZEB2**	**NFIA** GATAD1 FOXN3
Gokce *et al.* 2016	Striatal macrophage	**MAF FLI1 KLF4 ATF3 IRF1**	JUN **TCF4 IRF8**
Scialdone *et al.* 2016	Embryonic blood progenitor	**GATA2 FLI1 STAT5B HHEX TAL1**	**YBX1** UBTF ZFP568
Scialdone *et al.* 2016	Embryonic primitive erythrocyte	**KLF1 GATA1 ZFPM1 STAT5B MYB**	HMGB3 SUB1 GM6104
Scialdone *et al.* 2016	Embryonic early mesodermal progenitor	**MESP1 MSX2 FOXF1** LEF1 PEG3	**FOXH1** HMGA2 SALL4
Scialdone *et al.* 2016	Embryonic visceral endoderm	**HNF4A LHX1 GATA4** TCF7L2 **SMAD1**	HMGB3 **EOMES** FOXP4
Joost *et al.* 2016	Hair interfollicular epidermis differentiated cell II	**GRHL1 IRF6** KDM5B **SP6** YBX3	**CEBPA KLF4 JUN**
Joost *et al.* 2016	Hair follicle inner bulge cell	TEAD2 HOXC13 **FOXP1 FOXC1 MITF**	YBX3 KLF6 KDM5B
Cohen *et al.* 2018	Lung B cell	**PAX5** SPIB **EBF1 PML SP110**	AES DRAP1 MEF2D
Cohen *et al.* 2018	Lung T cell	BRD7 DRAP1 NFE2L2 **IKZF2** JUND	MTA2 SSBP3 TAF10
Cohen *et al.* 2018	Lung macrophage I	**IRF8 XBP1 IRF5 STAT6 LITAF**	**FLI1** EDF1 **SF1**
Cohen *et al.* 2018	Lung neutrophile	**CEBPE** NFE2 ARID5A **JDP2** BCL3	IRF1 CHD7 BRD4
Cohen *et al.* 2018	Lung natural killer cell	**TBX21 IKZF3 GATA3 RUNX3** TCF7	CITED2 SF1 HNRNPD
Cohen *et al.* 2018	Lung Mast cell	**GFI1 GATA2** MYB **MITF** RUNX3	BRD4 BAZ1A MEIS1
Segerstolpe *et al.* 2016	Pancreatic alpha cell	**MAFB** ISL1 **NEUROD1**	PCBD1 HMGN3 RAN
Segerstolpe *et al.* 2016	Pancreatic beta cell	**NKX6**-**1** CNBP CSDE1 CCT4	PSMC5 **NEUROD1 MAFB**
Segerstolpe *et al.* 2016	Pancreatic delta cell	**HHEX** PSIP1 **ISL1** EHF CBFB	ENO1 SAP18 HMGN3

Previously experimentally validated identity TFs are marked in bold. See Supplementary Table S1 for literature evidence for identity TFs and Supplementary Table S2 for the complete list of identified synergistic identity cores.

It is worth noticing that cell subpopulation identity TFs depend on the level of cell specialization when comparing different subpopulations within the population (6). For example, in the case of cell types (e.g. erythrocytes and hepatocytes), the identity TFs are determined by the comparison of the transcriptomes for these clearly different cell types. This, however, becomes more subtle in the case of subtypes of a same cell type (e.g. subtypes of dopaminergic neurons) due to their developmental closeness, reflected in the similarities of their transcriptional profiles.

Here, we postulate that subpopulation identity is determined by a unique combination of TFs that are mainly specifically expressed in a target subpopulation, but also TFs that are not specifically but more broadly expressed in the entire population, which together exhibit high synergy at the transcriptional level. We refer to this TF combination as a *synergistic identity core*. Experimental evidence in various systems shows that several TFs are necessary to operate together in order to maintain a cellular phenotype (7–10). In particular, in the case of embryonic stem cells, cooperation of TFs forming a transcriptional core involving Pou5f1, Sox2 and Nanog is crucial for controlling pluripotency (8). Further, combinatorial binding of specific TFs to enhancers gives rise to a synergistic activity, which in turn is crucial for robust and specific execution of transcriptional programmes of development (9). Finally, it has been shown that the combinatorial expression of a relatively limited number of TFs is enough to establish, and potentially convert, cell identity (10).

The term ‘*synergy*’ is used here to describe an emergent property of a complex system manifested in the cooperative action of two or more of its elements that results in a different, or stronger, effect than the sum of the elements acting in separation. In other words, a system is *synergistic* if its overall behaviour cannot be deduced from the sum of the behaviours of its individual parts. In this study it is the interactions among more than two TFs, which cannot be inferred by their pairwise interactions alone. Building on this view, we consider an information theory-based measure of multiple correlation to capture synergy among a set of TFs. Based on this, we devise a computational method that searches for a combination of TFs that form a synergistic identity core of a given target subpopulation with respect to the other subpopulations (referred to as background subpopulations) present in a considered cell population. The context (i.e. background subpopulations) is important to consider, as depending on, for example, whether the identity core TFs for a cell type or for a cell subtype are sought, the result may be different. Moreover, the background subpopulations enable us to define which TFs are specifically expressed in the target subpopulation and which are more broadly expressed in the entire population. Our method uses only single-cell RNA-seq data, and does not require additional prior knowledge or inference of gene regulatory networks. In particular, the latter constitutes a desirable feature, as inferred gene regulatory networks are incomplete and often fail to capture multiple direct and indirect gene interactions.

By applying our method to a collection of datasets available in literature, we show that our computational approach can correctly recapitulate experimentally validated known identity TFs among those forming the predicted synergistic identity cores. Moreover, by considering examples of TF-mediated cell conversion experiments reported in literature, we show that our method can identify core TFs whose perturbations have been shown to convert one cell type into another, including mouse embryonic fibroblasts into oligodendrocytes, embryonic and postnatal mouse fibroblasts into astrocytes, and mouse embryonic fibroblasts into haematopoietic progenitors. Importantly, we show that the detected synergistic identity cores cannot be obtained by simply considering pairwise mutual information between the constituent TFs. This result indicates that indeed the identity TFs need to be considered in combination as a whole in order to capture the emergent property of synergy arising from multiple interactions among them.

In conclusion, we argue that the knowledge of the synergistic identity core that is responsible for maintaining the identity of a given target subpopulation with respect to background subpopulations has important implications for cell conversion and regenerative medicine. In particular, due to the synergistic character of identity cores, perturbation of only a few key TFs should be sufficient to trigger a cell conversion from a target subpopulation to any of the background subpopulations. In this way, our computational method can potentially be used to improve efficiency and fidelity of cell conversion.

## MATERIALS AND METHODS

### Principles and outline of the computational method

Comparison of gene expression profiles of cell subpopulations enables the identification of target subpopulation transcriptional cores formed by a specific combination of TFs. We postulate that the target subpopulation transcriptional core is primarily composed of a synergistic combination of TFs that are uniquely expressed in the target subpopulation but not in background subpopulations. In addition, other TFs, which are also expressed in background subpopulations and contribute to the overall synergy, can be part of the core. Thus, the target subpopulation transcriptional core is characterized by a unique synergistic combination of those TFs.

Our computational method for the identification of target subpopulation transcriptional cores proceeds as follows. In the first stage (Stage I), candidate TFs for identity TFs are prefiltered: TFs that both have the highest expression ratio in the target subpopulation and are specifically expressed in the target subpopulation but not in the background are selected. In the second stage (Stage II), all subsets of the target subpopulation-specific TFs of sizes ranging from three to five are considered and the most synergistic subsets are identified. Finally, in the third stage (Stage III), in order to account for the possibility that the identity core can consist also of TFs that are non-specifically expressed in the target subpopulations, the set of TFs found in Stage II is extended by all possible combinations of size three among such TFs. The most synergistic extended set is reported as the synergistic identity core for the target subpopulation.

The particular choice of the sizes of subsets considered by our method is made to facilitate exhaustive computations for all subsets and by the fact that the transcriptional identity core is formed by a relatively limited number of TFs (10). Moreover, as the transcriptional core is assumed to be composed primarily of a synergistic combination of target subpopulation-specific TFs, sets of these TFs are considered first. Once most synergistic combinations of these TFs are found, they are further extended with TFs that are not target subpopulation specific, and can increase the overall synergy of the complete cores. We present the details of each of the three stages of our method in the following sections.

### Stage I: Identifying sets of target subpopulation-specific TFs

In order to determine the set of TFs that have the highest expression ratio in the target subpopulation, the following procedure is employed. We denote the set of all TFs present in the considered single-cell RNA-seq dataset as $TF$. First, for each $tf$ in $TF$ its *expression ratio* is computed (i.e., the ratio of the number of cells of the target subpopulation, in which the considered TF is expressed to the total number of cells of the target subpopulation). We denote this value $expressio{n_{ratio}}( {tf} )$. A cell is considered to express a given TF if the TF’s expression value in this cell is equal to or above an expression threshold value that is specific to the target subpopulation dataset as reported in the ‘Single-cell RNA-seq data’ section. We select TFs with expression ratios greater or equal to the eighth sample decile (i.e. the expression ratio value such that 80% of all considered TFs have lower expression ratio value). In addition, as this 8th sample decile set is often too large for subsequent exhaustive computation of synergy, we further reduce this set by taking the TFs with top 150 lowest coefficient of variations (CVs) in this set. The CV is used, since the expressions of identity TFs are often tightly regulated (i.e., low variation) in many cells of the target subpopulation. More formally, the prefiltered set of TFs is defined as

\begin{eqnarray*} {{\rm prefiltered}_{{\rm TFs}}} &=& \lbrace{ tf \in TF{\rm{|}}\ {{\rm expression}_{{\rm ratio}}}\left( {tf} \right)}\\ &&{\ge d{8_{{\rm expr}}} \cap {\rm rank}\left( {{\rm CV}\left( {tf} \right)} \right) \le 150 \rbrace} , \end{eqnarray*}

where ${\rm CV}$ is the ratio of the sample standard deviation to the mean, and $d{8_{expr}}$ is obtained with the *quantile* function of package *stats* version 3.5.1 of R with type = 3 and probs = 0.8 applied to the expression ratios of all TFs).

The set of TFs that are specific to the target subpopulation is determined in the following way. Let $n$ be the number of cells in the target subpopulation and $m$ be the number of TFs present in the considered single-cell RNA-seq dataset. Further, let $t{f_i}$ be the i-th TF, where $i$ is in the range $[ {1..m} ]$, and let ${c_j}$ be the j-th cell of the target subpopulation, where $j$ is in the range $[ {1..n} ]$. First, for each $t{f_i}$ and each cell ${c_j}$ of the target subpopulation, the expression vector ${v_{t{f_i},{c_j}}}$ is considered, where ${v_{t{f_i},{c_j}}}[ 1 ]$ is the expression value of $t{f_i}$ in cell ${c_j}$ and the remaining elements contain the expression values of $t{f_i}$ in all individual cells of the background subpopulations put in some arbitrary but fixed order. All expression vectors are of the same length equal to one plus the number of cells in the background subpopulations. By construction, for any given $t{f_i}$ its $n$ expression vectors differ only in the first element. Second, we normalize each expression vector by dividing each of its elements by the sum of all its element values. A TF that is specific for the target subpopulation in the ideal case would have a normalized expression vector that has 1 in the first element and 0’s in all the other elements, which corresponds to the situation where the TF is expressed in the respective cell of the target subpopulation and has zero expression value in all cells of the background subpopulations. We use ${v_{ideal}}$ to denote the ideal normalized expression vector. For each normalized expression vector ${v_{t{f_i},{c_j}}}$, we evaluate its proximity to ${v_{ideal}}$ by considering the two vectors as discrete probability distributions and by calculating the JSD value defined for any two discrete probability distributions $P$ and $Q$ as

\begin{equation*}{\rm JSD} \left( {P,Q} \right) = \frac{1}{2}\ {D_{KL}}\left( {P,M} \right) + \frac{1}{2}{D_{KL}}\left( {Q,M} \right),\end{equation*}

where $M\ = \frac{1}{2}\ ( {P + Q} )$ and ${D_{KL}}$ is the Kullback–Leibler divergence, i.e.

\begin{equation*} {D_{KL}}\ \left( {P,Q} \right) = \mathop \sum \nolimits_i P\left[ i \right]\log\frac{{P\left[ i \right]}}{{Q\left[ i \right]}} \end{equation*}

Once the JSD values are calculated for normalized expression vectors for all $prefiltere{d_{TFs}}$ and all cells in the target subpopulation, we compute the summed JSD value $summedJSD$ for each TF over all cells in the target subpopulation as

\begin{equation*}{\rm summedJSD} \left( {tf} \right) = \mathop \sum \nolimits_j^n {{\rm JSD}_j}\left( {tf} \right). \end{equation*}

Since this JSD value measures the closeness of the observed gene expression profile to the ideal gene expression profile, the lower the value, the higher the specificity of the expression of $tf$ to the target subpopulation. Following the previous bulk JSD approach by (4), we rank the TFs by these $summedJS{D_{TF}}$ values, and select as target subpopulation-specific TFs those which have the top ten lowest ${\rm summedJSD_{TF}}$ values:

\begin{equation*}{{\rm SPEC}_{{\rm TFs}}} = {\rm{\{ }}tf \in TF{\rm{|}}\ {\rm rank}\left( {{{\rm summedJSD}_{TF}}\left( {tf} \right)} \right) \le 10\} .\end{equation*}

In addition, TFs that are non-specifically expressed in the target subpopulation and have the top 50 highest expression ratio values are selected from $prefiltere{d_{TFs}}$, i.e.,

\begin{eqnarray*} {{\rm nonSPEC}_{{\rm TFs}}} &=& \lbrace tf \in TF{\rm{|}}\ {\rm rank} \\ && \times \left( {{{\rm prefiltered}_{TFs}} \setminus {{\rm SPEC}_{TFs}}} \right) \le 50 \rbrace. \end{eqnarray*}

The top 50 is set, as above this value, the exhaustive computation of MMI for all subsets of three TFs (see Stage III) becomes too long or crashes due to the out-of-memory error.

The aim with the JSD operation is to identify TFs that both have the highest expression ratio in the target subpopulation and are target subpopulation-specific. For the latter, as described above, we first consider the JSD values of normalized expression vectors. This idea is based on the approach proposed in (4) for identifying candidate TFs that control cell identity. Therein, the TF expression-specificity score based on the JSD measure was introduced. The JSD measure was applied to TF expression vectors with elements containing normalized expression values for individual cell types measured in bulk experiments. Top ten TFs with the lowest scores were then taken as candidate identity TFs. However, in our case, single-cell data are considered. Therefore, we apply the JSD measure to TF expression vectors containing normalized single-cell expression values, and consider each cell of the target subpopulation separately. Then, we combine the obtained result to compute the ${{\rm summedJSD}_{TF}}$ value for the TFs. Contrary to the approach in (4), we do not use the JSD measure for the final identification of core TFs but consider it only for the pre-selection of TFs that are given as input to the subsequent, main analysis.

### Stage II: Identifying the most synergistic subset of target subpopulation-specific TFs

Given the set of candidate TFs specifically expressed in the target subpopulation, ${{\rm SPEC}_{TFs}}$, a search for a subset with the highest synergy is performed. To quantify synergy, we apply the information theory-based measure of the multivariate mutual information (${\rm MMI}$) (also known as co-information) (11) to a set of random variables $S\ = \{ {{X_1},{X_2}, \ldots ,{X_k}} \}$ which can be expressed in terms of Shannon's entropies ($H$) as follows:

\begin{equation*} {\rm MMI} \left( S \right) = - \mathop \sum \nolimits_{T \subseteq S} {\left( { - 1} \right)^{\left| T \right|}}H\left( T \right), \end{equation*}

where $T$ is a subset of $S$ and $| T |$ denotes the cardinality of $T$. For example, in the case of three variables, $X$, $Y$ and $Z$, the equation can be expanded as

\begin{eqnarray*} {\rm MMI} \left( {X;Y;Z} \right) &=& H\left( X \right) + H\left( Y \right) + H\left( Z \right) - H\left( {X,Y} \right) \nonumber \\ &&- H\left( {X,Z} \right) - H\left( {Y,Z} \right) + H\left( {X,Y,Z} \right), \end{eqnarray*}

where $H( {X,Y} )$ denotes the joint Shannon's entropy of $X$ and $Y$, and so on. MMI quantifies synergistic interactions among random variables. It is symmetric with regard to all variables considered (i.e. it treats all variables in the set equally); none of them is distinguished, and the value can be positive or negative. Hence, where considered as a measure of synergy, the following interpretation of MMI has been assumed: negative values imply a synergistic interaction among the variables and positive values are considered to indicate redundant information among the variables (11,12).

Each TF is considered as a random variable whose realizations are its expression values in the individual $n$ cells of the target subpopulation. To compute Shannon's entropies in the above equation, the respective gene expression values in the target subpopulation are first log_10_-transformed, where zero expression values of a TF are replaced with ‘1’ prior to the transformation. After the transformation, the expression values for each TF in the target subpopulation are discretized by binning the values into a number of bins determined by applying the Freedman-Diaconis rule implemented in the *nclass.FD* function of the version 3.5.1 R package to the log10-transformed expression values of the TF across the entire population. Note, due to the difference in the implementation of the *nclass.FD* function, R package version ≥3.5 is required. The bin size is set to the value returned by the *nclass.FD* function +1. Since the number of bins varies with TFs, to facilitate fair comparisons, Shannon's entropies are normalized by the theoretical maximum Shannon's entropy of the respective bin size as

\begin{equation*}{H_{norm}} \left( S \right) = \frac{{H\left( S \right)}}{{{H_{max}}\left( S \right)}}\ = \frac{{H\left( S \right)}}{{log2\prod r}}\ ,\end{equation*}

where $r$ is a vector of bin sizes for variables in $S$.

As MMI is a measure that quantifies synergy among $k$ variables with respect to a subset of $k - 1$ variables, we propose an algorithm that searches for the most synergistic core in an incremental way. We base our search for the most synergistic core on the following reasoning. Adding a new, single element to a strongly synergistic set of size $s$ should result in an enlarged set that is still significantly synergistic compared to all sets of size $s + 1$. This should remain true, at least to some extent, irrespective of what the new element is. Therefore, many of the sets formed in this way should rank high according to their MMI values among sets of size $s + 1$.

The search algorithm consists of three steps. First, all subsets of ${{\rm SPEC}_{{\rm TFs}}}$ of size three are ranked by MMI. Among the cores with negative MMI values, the top ten most synergistic cores are selected. Second, each of the selected cores of size three is extended by adding each of the new TFs from $SPE{C_{TFs}}$ (i.e., $SPE{C_{TFs}} \vee - 3$). Third, among the newly formed cores of size four with negative MMI values, the top ten most synergistic cores are selected and extended by adding each of the new TFs from $SPE{C_{TFs}}$, (i.e., $SPE{C_{TFs}} \vee - 4$). This iteration continues until either an iteration in which sets of the pre-specified maximum core size are considered has been executed. or the current iteration ends with an empty family of sets chosen for the next iteration. In our analyses we set the maximum core size to five.

### Stage III: Adding non target-subpopulation specific TFs

The core returned by Stage II is obtained by considering TFs only in $SPE{C_{TFs}}$, that is, TFs which are target subpopulation specific. In Stage III the method takes into account our proposition that a synergistic identity cores contains TFs that are expressed in the target subpopulation but are not specific to it. For this, all extensions of the core from Stage II by all subsets of size three of $nonSPE{C_{TFs}}$ are considered. The set consisting of TFs obtained in Stage II and the newly added TF triplet, which gives rise to the most synergistic, overall MMI, is returned as the synergistic identity core of the target subpopulation with respect to the considered background subpopulations.

### Enrichment test for identity TFs

The statistical enrichment of identity TFs with experimental evidence (known identity TFs) is computed by the hypergeometric test

\begin{equation*}P{\rm{\ }}\left( {X = k} \right) = \frac{{\left( {\begin{array}{@{}*{1}{c}@{}} K\\ k \end{array}} \right)\left( {\begin{array}{@{}*{1}{c}@{}} {N - K}\\ {n - k} \end{array}} \right)}}{{\left( {\begin{array}{@{}*{1}{c}@{}} N\\ n \end{array}} \right)}}\ \end{equation*}

where $K$ is the number of known identity TFs that are present in the set of specifically expressed TFs (i.e. $SPE{C_{TFs}}$), $k$ is the number of known identity TFs that are present in the subset of $K$ that is present in the synergistic identity cores, $N$ is the number of $SPE{C_{TFs}}$, and $n$ is the number of TFs from $SPE{C_{TFs}}$ that are present in the synergistic cores.

### Single-cell RNA-seq data

Single-cell RNA-seq data used in this study are obtained for the following biological systems; the mouse datasets for lung, striatum, cortex and hippocampus, intestine organoids, hair follicles and gastrulating embryo, and the human dataset for pancreas. The reference to each dataset is described in Supplementary Table S2. To facilitate the method benchmarking, we do not use datasets that do not have more than five subpopulations of well-defined cell (sub) types, or whose subpopulations do not come from the same tissue or are the derivatives of artificial cellular reprogramming. We use the same subpopulation classifications defined in the original studies. We do not reprocess each raw data and same gene expression values that were used in the original studies are also used in this study. TFs are considered ‘expressed’ if their expression values are ≥1 in FPKM/RPKM/TPM/UMI. TFs below these thresholds are considered ‘not expressed’.

## RESULTS

We postulate that the identity of a cell subpopulation is relative, and is determined by the background subpopulations, and therefore our computational method discerns a synergistic identity core of the given target subpopulation with respect to background subpopulations. Furthermore, we propose that a synergistic identity core of a subpopulation is mainly composed of TFs that are specifically expressed in a target subpopulation, but also of TFs that are not specifically but more broadly expressed in background subpopulations which contribute to the overall synergy of the identity core. Notably, by considering combinations of TFs rather than individual TFs, our method searches for an identity core composed of TFs that possess a synergistic co-expression pattern, which reflects direct and indirect tight regulatory interactions between the TFs. In this way, the approach aims to take into account synergistic transcriptional regulation, including protein-protein interactions between transcriptional co-factors and cooperative binding of TFs to promoter/enhancer regions of target genes (9).

**Figure 1.**
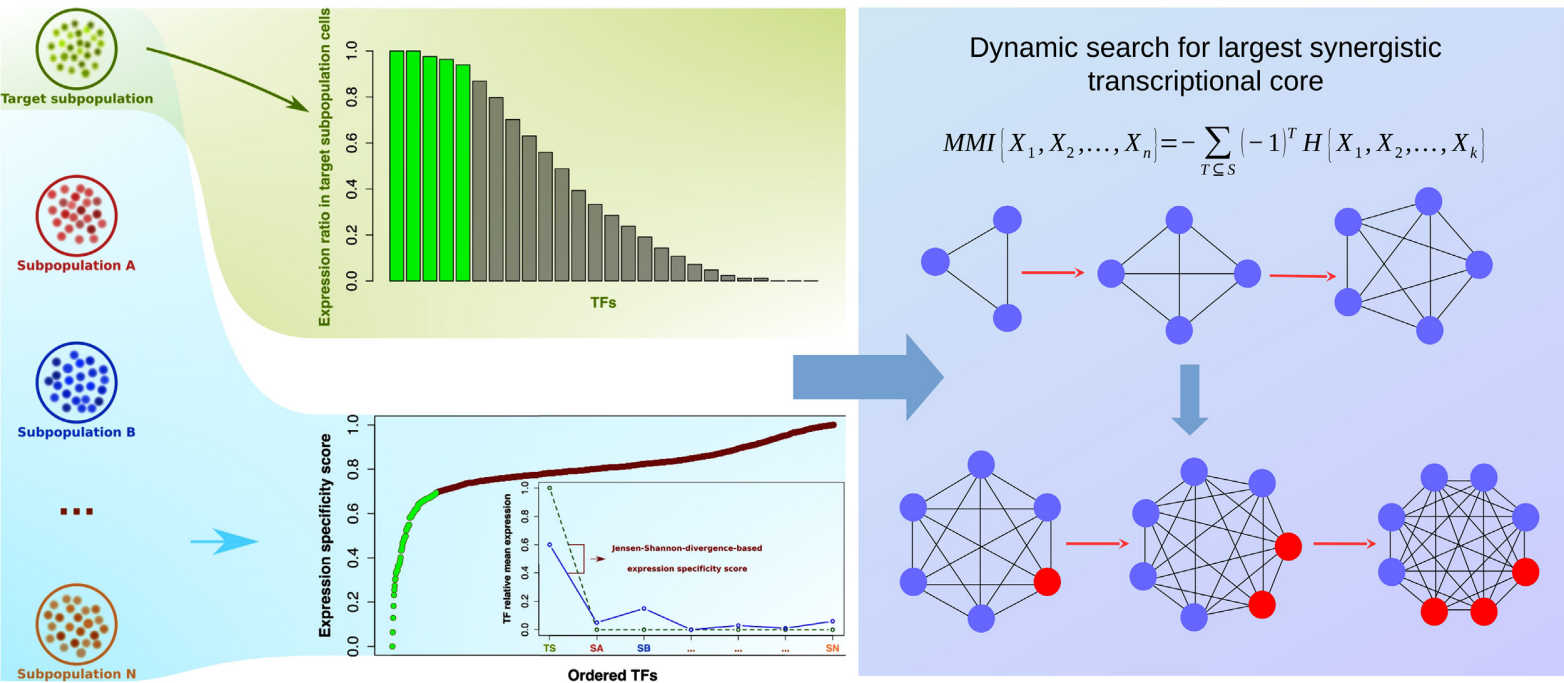
Schematic overview of method. For each target subpopulation, a set of TFs that are specifically expressed in the target subpopupulation (specific TFs) is identified based on expression ratio and JSD with respect to background subpopulations (left panel). Then most synergistic subset of specific TFs is sought by computing multivariate mutual information (MMI). Once a most synergistic core is found, it is extended by computing MMI with TFs expressed in both target and background subpopulations (non-specific TFs). Blue circles and red circles in the right panel indicate specific TFs and non-specific TFs, respectively.

As input, the proposed method only requires single-cell RNA-seq data of the target subpopulation and a number of background subpopulations. By the fact that the method considers synergistic combinations of TFs rather than individual TFs, it is capable of capturing the core identity TFs of the target subpopulation even in the cases where background subpopulations are closely related to the target one without any additional prior knowledge or gene regulatory network inference. The overview of the method is presented in Figure 1.

### Method evaluation

To assess the performance of our method, we calculated the percentage of target subpopulations for which at least one of the predicted core TFs has been experimentally validated to define the identity of the target subpopulation. We applied our method to eight published mouse and human single-cell RNA-seq datasets, consisting of 88 subpopulations (Supplementary Table S2), for which literature evidence of identity TFs is available (see also Methods). Here, by literature evidence we mean experimental studies where TFs were shown to disrupt the phenotype of, or the differentiation into, the target cell type upon silencing, or they were shown to induce reprogramming/differentiation into the target cell type upon overexpression. We did not consider hight/unique gene expression states alone as experimental evidence for identity TFs. Our method was able to predict at least one such TF for 74% of the considered target subpopulations (Supplementary Table S2). We considered this simple criterion due to the fact that the state-of-the-art knowledge of experimentally validated identity TFs is by no means complete. In fact, predicted TFs without experimental evidence constitute novel candidate TFs for experimental follow-ups. A list of identity TFs with literature evidence in each subpopulation considered in this study is presented in Supplementary Table S1.

We also evaluated the effectiveness of transcriptional synergy in selecting known identity TFs from target subpopulation-specifc TFs (i.e., $SPE{C_{TFs}}$). For this purpose, we performed a hypergeometric test to compute the enrichment of known identity TFs in the target subpopulation-specific part of the synergistic identity core with respect to the entire set of subpopulation-specific TFs (see Methods). As the synergistic identity core for each subpppulation consists of at most five of those TFs, the meaningful statistical enrichment is difficult to compute for each subpopulation. Therefore, we pooled all the 88 subpopulations, and computed the identity TF enrichment. This resulted in a *P*-value equal to 2.617e–11, which is statistically significant and unlikely to become insignificant due to small variations in the composition of the synergistic identity cores. Hence, we conclude that subpopulation identity tends to be maintained by TFs that exhibit high transcriptional synergy and the computation of synergistic cores can selectively further reduce the candidate TF space from the $SPE{C_{TFs}}$ sets.

To investigate to what extent our method is sensitive to the choice of core size, we performed a sensitivity analysis of our method with respect to the maximum size of the target subpopulation-specific part of synergistic identity cores (maximum core size). For each maximum core size ranging from four to seven, synergistic identity cores were identified and the statistical enrichment for known identity TFs was computed, as described above. The *P*-values were 3.003e–3, 1.093e–5 and 1.576e–4 for the maximum core sizes four, six and seven, respectively, indicating that synergistic identity cores are enriched with known identity TFs at these maximum core sizes too. This analysis also shows that maximum core size five is the optimal in terms of the enrichment of known identity TFs in synergistic identity cores. The entire list of identified synergistic identity cores at each maximum core size is presented in Supplementary Table S3. In addition, in order to assess the robustness of the method to the maximum core size, we computed the percentage of overlapping TFs with those obtained with maximum core size five. When the maximum core size was set to four, six and seven, the overlap of entire synergistic identity cores (including three additional non-specific TFs) was 84%, 88% and 86%, respectively (Supplementary Figure S1), which is roughly equivalent to a one TF difference out of eight total TFs in each synergistic identity core. This result suggests that our method is not substantially sensitive to the maximum core size of the target subpopulation-specific part of synergistic cores. Therefore, in order to comply with the biological knowledge that identity cores usually consist of a handful of TFs and given that the highest, statistically significant enrichment for known identity TFs was obtained, we think the choice of maximum core size five is legitimate. Note, the sensitivity of the set size for non-specific part of synergistic cores could not be evaluated, as the set size larger than three resulted in memory allocation errors (gigabytes) due to too many combinations of TFs for computing MMI.

Next, we evaluated whether pairwise MMI (i.e. mutual information) was capable of revealing all the interactions between the TFs constituting the synergistic identity cores reported by our method. The result showed that the majority of pairs of core TFs did not appear among the top pairs with the highest mutual information values (Supplementary Figure S2). In other words, the identified synergistic identity cores could not be obtained by simply considering pairwise mutual information between the constituent TFs. Therefore, all core TFs need to be considered in combination in order to capture their emergent property of synergy arising from multiple interactions among them. Moreover, we investigated whether pairs of all known identity TFs in synergistic identity cores (Supplementary Table S2) were characterized by high mutual information and the result is shown in the form of histograms (Supplementary Figure S3). It indicated that in the majority of cases known identity TFs did not form pairs with high mutual information values. Consequently, constructing identity cores from pairs of TFs with high mutual information values would unlikely result in cores enriched with known identity TFs. In conclusion, the obtained results provide another support for our notion that the determination of identity cores requires the consideration of combinations of TFs taken as a whole rather than individual pairs of TFs.

To further evaluate the performance of our method, we compared it with the performances of other approaches presented in literature. First, we considered a method that finds candidate identity TFs for bulk cell/tissue types using the JSD measure (4). In brief, this approach ranks TFs considered individually by the closeness of their expression pattern to the ideal one which represents the condition of a TF to be expressed in the target subpopulation and in none of the background subpopulations. The closeness is quantified by the so-called *cell-type-specificity score*, the value of JSD applied to an observed expression vector consisting of normalized bulk expression measurements of a TF in the target and background subpopulations and the ideal vector having 1 for the target subpopulation and 0’s everywhere else. As candidate identity TFs, the method simply reports the top ten TFs ranked by the cell-type-specificity score. Since in the original study the method is applied to bulk microarray gene expression data, we averaged single-cell RNA-seq data over each cell subpopulation prior to applying this method. It predicted at least one experimentally validated identity TF in 35% of the target subpopulations considered in this study (Supplementary Table S4). Compared to the 74% of our method, this result indicates that consideration of expression variability across individual cells within each subpopulation significantly improves the identification of TFs that maintain the identity of closely related cell subpopulations.

A thorough comparison of our method with the computational frameworks CellNet (3) and Mogrify (5) was difficult, as these tools do not allow users to provide single-cell RNA-seq data, they cannot be applied to cell/tissue types or subpopulations that are not contained within their hard-coded datasets. Truly, there was a marginal overlap between built-in datasets of these frameworks and pairs of initial and target subpopulations in each dataset considered in this study. Specifically, no pair of initial and target subpopulations appeared in the built-in datasets of CellNet, while Mogrify shared only a few of them (Supplementary Table S5). Known identity TFs were present among the reprogramming factors indicated by Mogrify for neural stem cells and endothelial cells.

### Known interactions in synergistic identity cores

The full lists of identified synergistic identity cores and experimental evidence for identity TFs are presented in Tables S2 and S1, respectively, and some notable examples are shown in Table 1. In addition, some of these known identity TFs in synergistic identity cores have been shown to interact with each other to carry out important functions related to respective subpopulation identities. For example, the embryonic blood progenitor subpopulation contained Tal1, Gata2 and Fli1 in its synergistic identity core. These TFs have been shown to form complexes via protein-protein interactions that stabilize their co-operative binding to DNA, and synergistically control and self-maintain this subpopulation identity (13). Therefore, this represents a known example where a synergistic interaction of TFs defines a cell subpopulation identity. In another example Zfpm1, Gata1, Klf1 and Myb, where Zfpm1 is known to physically interact with the other three to maintain erythroid cells (14–16), were found in the synergistic identity core of the embryonic primitive erythrocyte subpopulation. Importantly, these core TFs often did not show high pairwise mutual information among themselves (Supplementary Figure S3).

The synergistic identity core of the striatal oligodendrocyte included Sox10, Prox1, Olig2 and Zeb2, which are known to regulate each other and other downstream targets specific to this cell subpopulation (17–19). Stat6, Litaf, Irf8 and Irf5 were identified in the synergistic identity core of the lung macrophage I, which have been shown to interact with each other to define this cell identity (20,21). The synergistic core of the lung B cell contained Pax5 and Ebf1. These TFs are known to regulate each other, and are important for the maturation of this cell type (22–24). This synergistic identity core also included Spib, a downstream target of Pax5 in B cell differentiation (25). Neurod1 and Isl1 were contained in the synergistic identity core of pancreatic alpha cell, which have been shown to interact with each other in non-beta cells (26,27). The synergistic identity core of the cortical microglia contained Spi1 and Cebpa. These two TFs are crucial for the microglial functionalities (28–30), and are known to transcriptionally activate each other in the haematopoietic system (31,32). In addition, the synergistic identity core also contained Tfec and Fli1, which have been shown to be directly regulated by Spi1 (33,34).

Next, we specifically investigated the contributions of non-specific TFs to synergistic identity cores. Results revealed that in 36 subpopulations non-specific TFs contained at least one known identity TF, showing that these non-specific TFs also contribute to the synergistic identity core. Furthermore, extracting from MetaCore (35) literature validated known interactions between non-specific and specific TFs in each synergistic identity core showed the presence of at least one such interaction in 45 subpopulations (Supplementary Table S6). Moreover, among the 117 extracted interactions, 70 of them involved known identity TFs. Given 173 known identity TFs present in the synergistic identity cores of the 88 subpopulations and 687 total number of TFs in these synergistic identity cores, the interactions between specific TFs and non-specific TFs that involve known identity TFs exhibited a statistical enrichment (*P* = 4.304e–4). Thus, non-specific TFs had a high tendency to be involved in interactions with known identity TFs, possibly giving phenotype specificity to subpopulations.

For example, the synergistic identity core of the striatal oligodendrocyte contained Sox10 (specific) and Nfia (non-specific) and the cross-inhibition between these TFs has been reported to be important for oligodendrycyte specification (36). Mafb (specific) and Jun (non-specific) of the synergistic identity core of striatal microglia are known to form a complex to maintain its identity (37). In addition, the synergistic identity core of the striatal macrophage included Klf4, Atf3 and Irf1 (specific) and Tcf4 and Irf8 (non-specific). Klf4 is a direct target of Irf8 and they both regulate Atf3 in monocyte differentiation (21) and Irf1 has been shown to physically interact with Irf8 to enhance the Irf8 chromatin binding in macrophage (38), Sall4 (non-specific) was identified in the synergistic identity core of the epiblast, which is known to regulate Otx2 (specific) in embryonic stem cells (39). The synergistic identity core of the visceral endoderm subpopulation contained Lhx1, Gata4 and Smad1 (specific), which are known to interact with Eomes (non-specific) and regulate the development and maintenance of visceral endoderm (40–42). Dlx3 (specific) and Grhl3 (non-specific) were identified in the synergistic identity core of the interfollicular epidermal keratinocyte I. These two TFs have been shown to interact with each other, and regulate keratinocyte differentiation (43). Tcf7 and Bcl11b are known to regulate each other during T-cell differentiation (44,45) and both TFs were found in the synergistic identity core of the interfollicular epidermal T cell (specific and non-specific, respectively). The synergistic identity core of the pancreatic beta cell contained Nkx6-1 (specific) and Mafb (non-specific), which have been shown to interact with each other and are important for the maturation of beta cells (46). These observations support our notion that transcriptional identity cores are formed by the synergistic interactions of not only subpopulation specific TFs, but also subpopulation sepcific TFs and non-specific TFs.

In summary, the above results indicate that our method discerns synergistic identity cores that contain TFs that are known to maintain cell type/subpopulation identities and they capture known functional, potentially synergistic interactions between identity TFs.

### Cell conversion examples

There is evidence in literature for TFs that can convert one type of cells into another. These cell conversions usually do not occur during organism development, rather these TFs establish new cell identity upon artificial overexpression in the starting cells. For example, in the case of reprogramming mouse fibroblasts into astrocytes a synergistic effect between three TFs, Sox9, Nfia and Nfib, was observed (47). Therein, viral-mediated overexpression of the combination of these TFs mediated the successful derivation of the converted cells (i.e. iAstrocytes) that were functionally and transcriptionally comparable to native brain astrocytes. Consistently, the synergistic identity core of the striatal astrocyte contained Sox9 and Nfib, which have been more recently shown to rapidly and efficiently reprogramme human embryonic stem cells (ESCs) into functional astrocytes (48).

The three TFs identified in the synergistic core of the blood progenitor cells, Gata2, Tal1 and Fli1, have been shown to be able to reprogramme mouse embryonic fibroblasts into haematopoietic colonies (49). Indeed, these TFs have been shown to synergistically specify haematopoietic progenitors during mouse embryonic development (13). In another study it has been demonstrated that forced expression of zinc-finger nuclear protein Zfp521 in ESCs was able to establish the neural progenitor identity by directly activating early neural genes together with the endogenous co-activator Ep300 (50). Consistent with this study, the synergistic identity core of the striatal neural stem cells included Zfp521.

A study in (51) has shown that mouse embryonic fibroblasts can be successfully converted into induced oligodendrocyte precursor cells (iOPCs). The lineage of iOPCs is restricted to mature oligodendrocytes, as iOPCs generate neither neurons nor astrocytes under differentiation conditions *in vivo* (52). The reprogramming was achieved with the following combination of TFs: Sox10, Olig2 and Zfp536 (51). Additionally, it was shown that the induced expression of the found combination of TFs was capable of reprogramming mouse lung fibroblast into iOPCs. Furthermore, Sox10 alone has been shown to be able to convert satellite glia into oligodentrocyte-like cells (53). Consistent with these experimental studies, the synergistic identity cores identified by our method for both striatal oligodendrocyte and cortical oligodentrocyte contained both Sox10 and Olig2.

## DISCUSSION

In this study we introduced a new computational method for searching for a combination of TFs that form synergistic identity cores of target subpopulations in comparison with background subpopulations. We proposed that the identity of a cell subpopulation is relative and dependent on the background subpopulations. In addition, we hypothesized that the identity tends to be determined by a synergistic transcriptional core mainly composed of TFs that are specifically expressed in a target subpopulation, but also of TFs that are more broadly expressed in background subpopulations (i.e. non-specific to a target subpopulation). Commonly used network-based computational methods for determination of cell type-specific transcriptional cores rely on the construction of networks from pairwise correlated genes (3). Other existing methods are based on a statistical analysis of gene expression data (4), and do not take into account the synergistic interplay among identity TFs. Hence, these methods appear to be less efficient at capturing the experimentally observed cooperativity among these TFs that do not show high pairwise associations.

Our method takes as input only single-cell RNA-seq data, and is a network-free approach, which is a desired property considering the limitations of network reconstruction, such as incomplete prior knowledge and failure in distinguishing between direct and indirect gene interactions. We compiled a dataset comprised of different publicly available examples of cell populations, including in total 88 target subpopulations. By applying our method to this dataset, we showed that our approach was able to recapitulate experimentally validated identity TFs in majority of the subpopulations. These synergistic identity cores were enriched with not only known identity TFs, but also with known interactions that involve known identity TFs. Interestingly, pairwise mutual information between known identity TFs in synergistic identity cores were not always high, indicating that these TFs should be considered in combination to capture the emergent property of synergy, which arises from multiple interactions among them.

Further analysis showed that known cell conversion factors were present in synergistic identity cores identified by our method. Hence, we argue that identification of synergistic identity cores not only provides characterization of the target cell subpopulation identity, but can also facilitate the design of novel strategies for cell conversion between cell subpopulations. In particular, this methodology can be useful in identifying TFs capable of triggering cell conversions between novel subpopulations, including closely related ones that exhibit subtle differences in gene expression profiles. One limitation of the current implementation of the method is that the maximum size of the target subpopulation-specific part of synergistic identity cores is fixed to five. However, we observed that for five of the 88 subpopulations, there were more than five known identity TFs and not all of them can be identified in the synergistic identity cores if the maximum core size is set to five. Therefore, although we showed that this size is the optimal in terms of the enrichment of known identity TFs, the development of an algorithm for dynamically adjusting the maximum core size could improve the recall of the method.

Future extension of the current approach can be devised to select optimal combinations of core TFs, whose upregulation could increase cell conversion efficiency and fidelity. It would also be interesting to experimentally validate synergistic interactions among TFs in identified synergistic identity cores. Thus, our method can become a useful computational framework to guide experimental work in stem cell research and regenerative medicine.

## CONCLUSION

In this study, we postulated that identity of a cell subpopulation is relative, and is determined by the background subpopulations. Moreover, identity is determined by a unique combination of TFs that are mainly specifically expressed in a target subpopulation, but also TFs that are not specifically but more broadly expressed in background subpopulations, which together form a synergistic core at the transcriptional level. Based on this notion, we developed a novel computational method for the determination of synergistic identity cores of target subpopulations in a considered cell population. Application of the method to a large number of cell subpopulations demonstrated that it was able to recapitulate experimentally validated identity TFs in majority of the subpopulations. Importantly, our method is a network-free approach and does not rely on prior knowledge, and therefore it can be directly applied to any cellular system, including novel cell subpopulations for which single-cell RNA-seq data is available. Hence, this methodology can be useful for designing novel strategies for cell conversion, and in turn be a guidance to experimentalists in the field of stem cell research and regenerative medicine.

## DATA AVAILABILITY

The R and C++ scripts used to perform the computation of the synergistic identity cores are available at https://git-r3lab.uni.lu/satoshi.okawa/synergisticcore.

## Supplementary Material

Supplementary Data
