# Supplementary material for: A general computational approach to predicting synergistic transcriptional cores that determine cell subpopulation identities
Source: Nucleic Acids Res. 2019 Mar 1;47(7):3333–43. doi: 10.1093/nar/gkz147 (PMC6468312; doi:10.1093/nar/gkz147)
Supplement: Supplementary Data [file gkz147_supplemental_files.zip › suppl_captions_3.pdf]

Supplementary Figure 1: Percentage of overlapping TFs in identified synergistic identity cores. For each maximum size of the target subpopulation-specific part of synergistic identity core ranging from four to seven, the percentage of overlapping TFs with those obtained with core size five is computed. The mean and standard deviation over 88 subpopulations are plotted.

Supplementary Figure 2: Distributions of mutual information ranks for TF pairs in identified synergistic identity cores. For a given target subpopulation, all its pairs of TFs in $prefiltered_{TFs}$ are ranked by their mutual information values. Each histogram shows the counts of TF pairs from synergistic identity cores for respective target subpopulations. The binning is obtained by splitting the range from one to the total number of pairs of TFs into 20 intervals of equal length (i.e., each bin represents 5% of the total number of TF pairs).

Supplementary Figure 3: Distributions of mutual information ranks of known identity TF pairs. For a given target subpopulation, all pairs among its known identity TFs that are present in $prefiltered_{TFs}$ are ranked by their mutual information values. Each histogram shows the counts of pairs of known identity TFs present in respective target subpopulation synergistic identity core. The binning is obtained by splitting the range from one to the total number of pairs of TFs into 20 intervals of equal length (i.e., each bin represents 5% of the total number of TF pairs). If present, the left-most bar (i.e., one corresponding to the bin representing pairs with the highest mutual information values) indicates the rank number of the pair of known identity TFs with the highest mutual information value.

Supplementary Table 1: List of literature evidence for known identity TFs for each subpopulation. For each subpopulation, the literature evidence is collected for top ten specific TFs and three non-specific TFs in the identified synergistic identity core.

Supplementary Table 2: Identified synergistic identity core for each cell subpopulation. The 1st column describes the names of cell subpopulations identified in the original studies. The naming convention used in the original studies is kept. The 2nd column lists the specific-TF part of identified synergistic identity cores. TFs in bold have previous experimental evidence as identity TFs. The 4th column presents the number of experimentally validated identity TFs in the specific TF part of synergistic identity cores. The 5th column is the number of TFs in the specific TF part of synergistic identity cores. The 6th column is the number of experimentally validated TFs in the top ten specific TFs.

Supplementary Table 3: Identified synergistic identity core for each cell subpopulation with maximum core sizes for specific-part of core being four, six and seven. TFs in bold have previous experimental evidence as identity TFs. TFs that are not present in synergistic identity cores with maximum core size of five for specific part (Table S2) are indicated in red.

Supplementary Table 4: Top 10 TFs by JSD. JSD is computed for each TF in each cell subpopulation based on mean gene expression values following the approach proposed in [4]. Top 10 TFs (including ties) are taken as potential candidate TFs that define cell subpopulation identities (see Method evaluation for details).

Supplementary Table 5: Reprogramming factors predicted by Mogrify for cell subpopulations used in this study.

Supplementary Table 6: References (PMIDs) for interactions between target subpopulation-specific TFs and non-specific TFs of identified synergistic identity cores. Data was extracted from MetaCore [35].
