# Supplementary figures and images for: A general computational approach to predicting synergistic transcriptional cores that determine cell subpopulation identities
Source: Nucleic Acids Res. 2019 Mar 1;47(7):3333–43. doi: 10.1093/nar/gkz147 (PMC6468312; doi:10.1093/nar/gkz147)

# percentage of overlapping TFs with core size five

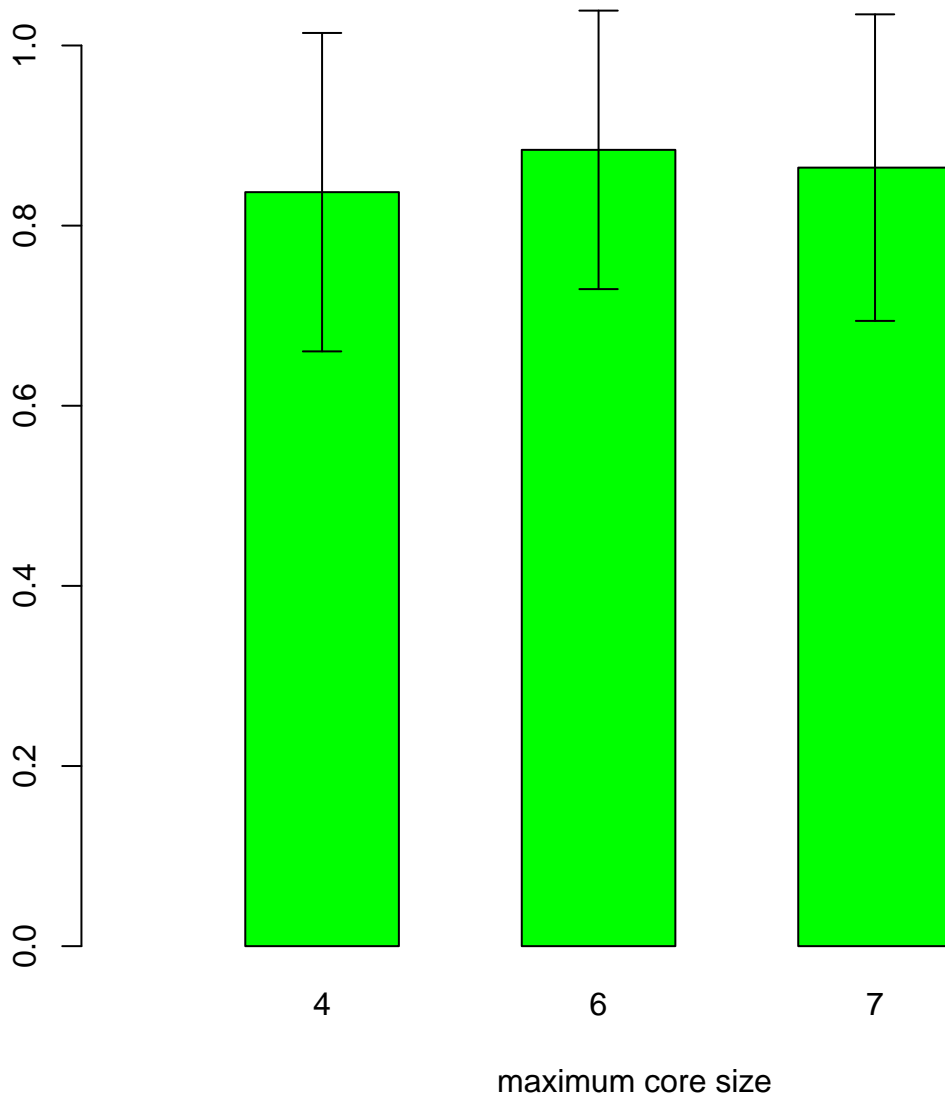

Supplement: Supplementary Data [file gkz147_supplemental_files.zip › Fig_S1.pdf]

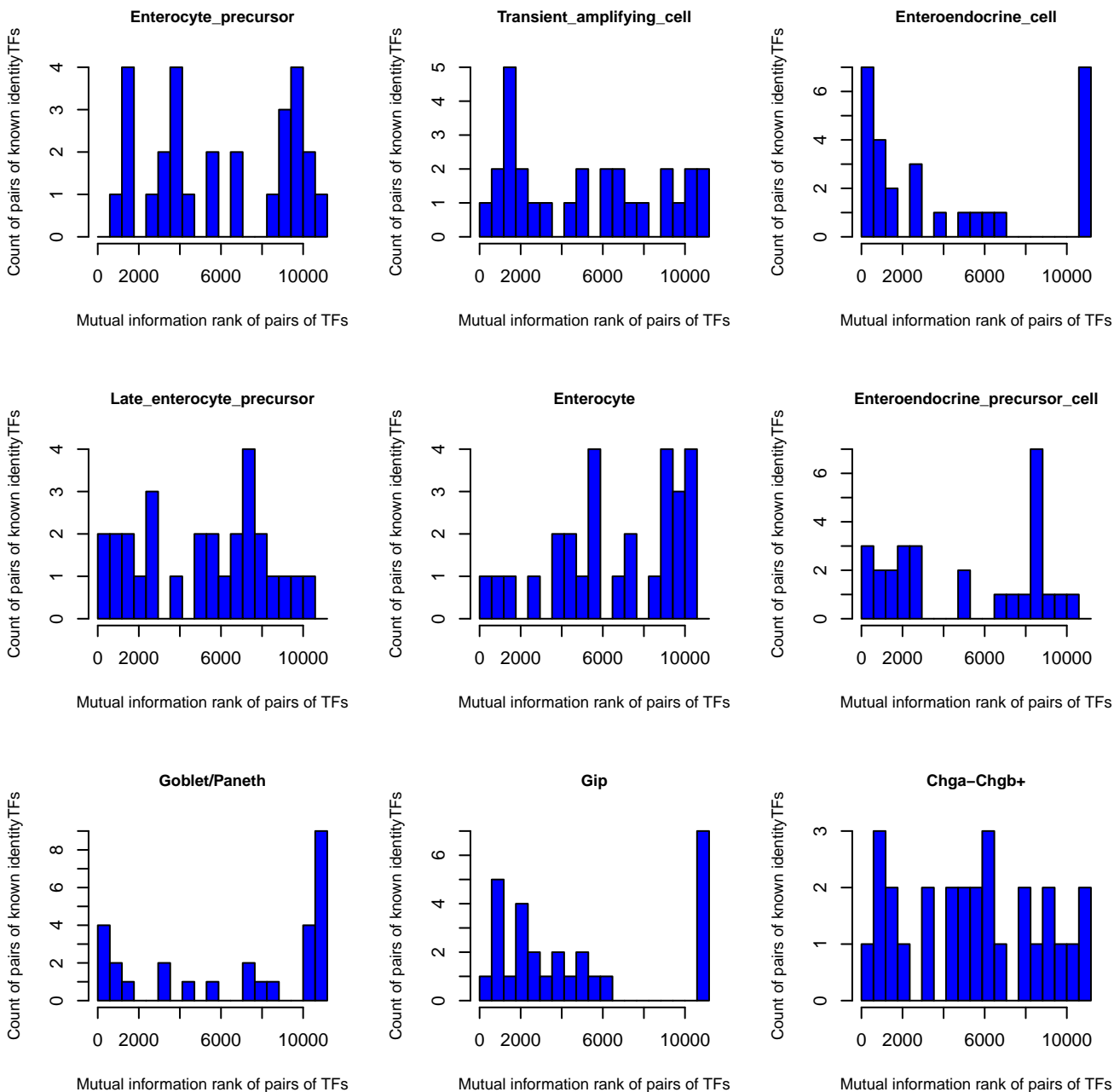

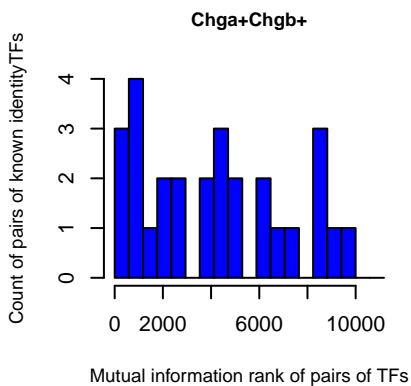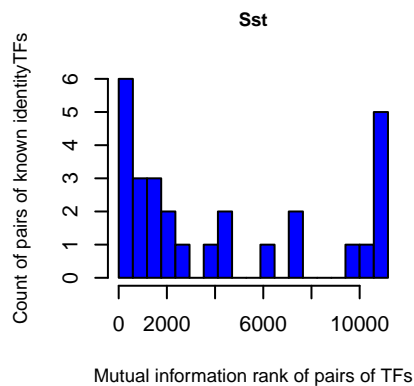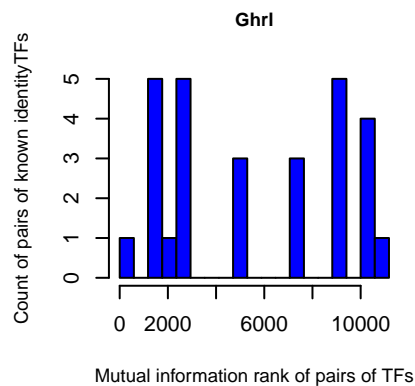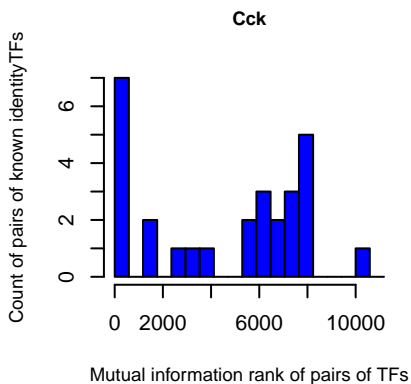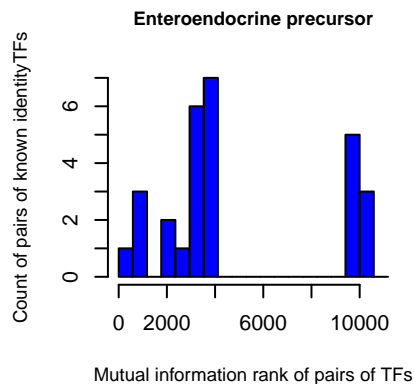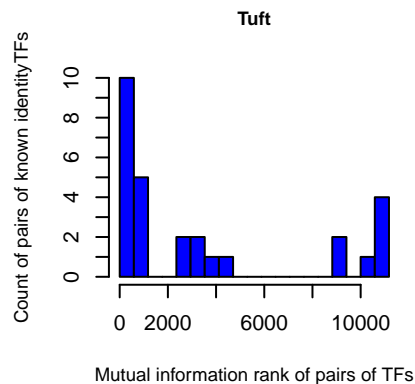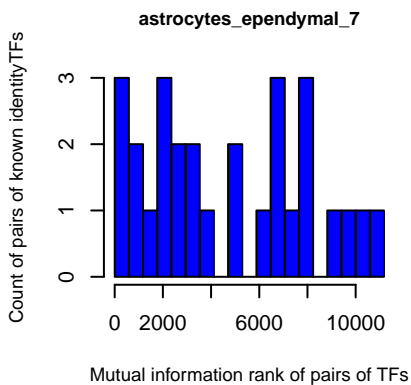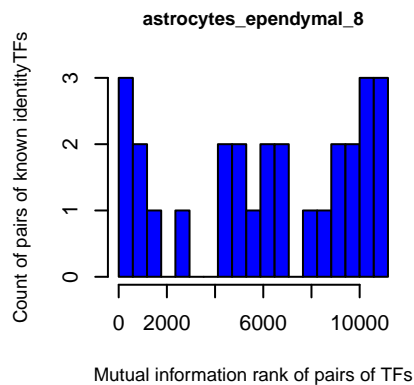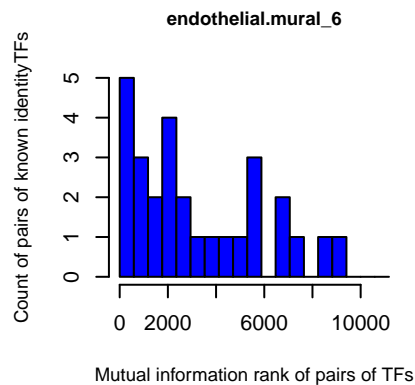

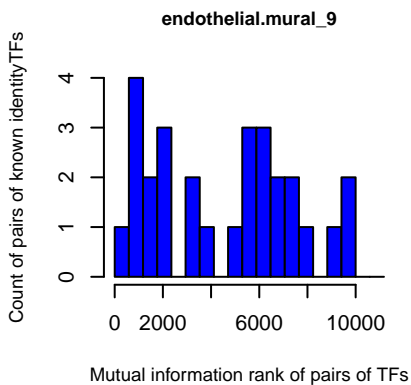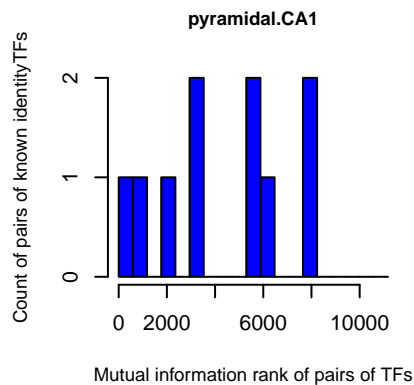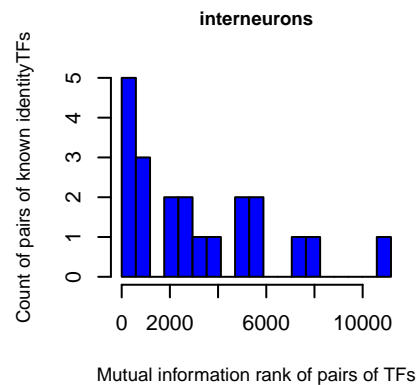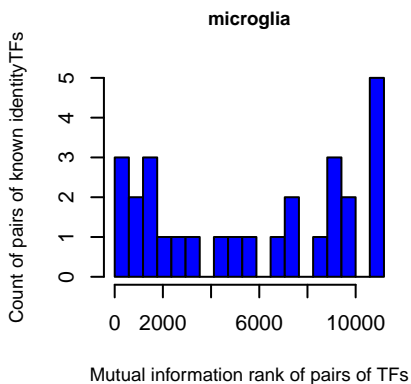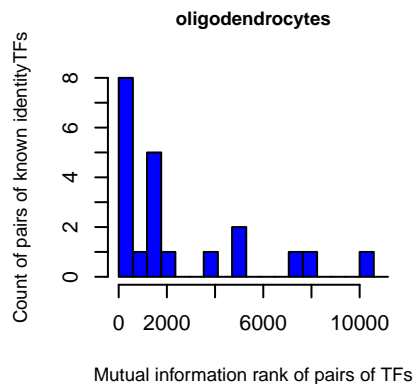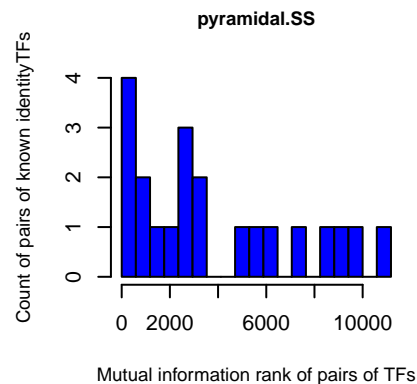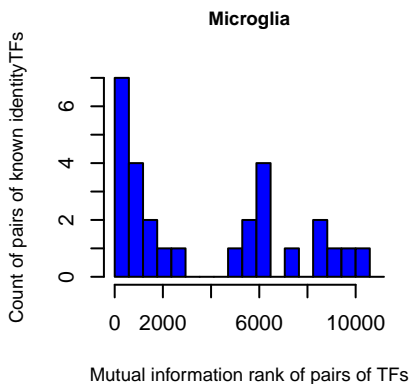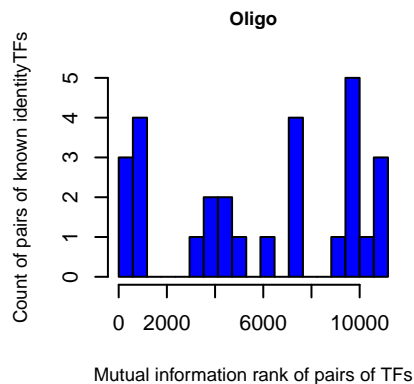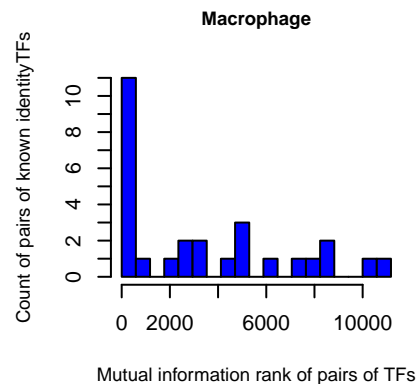

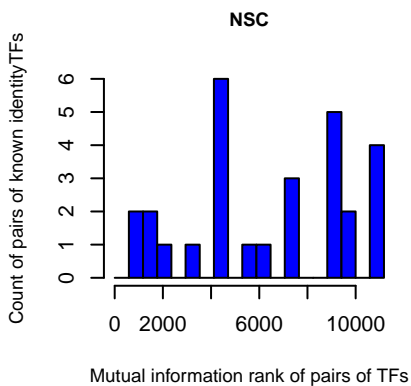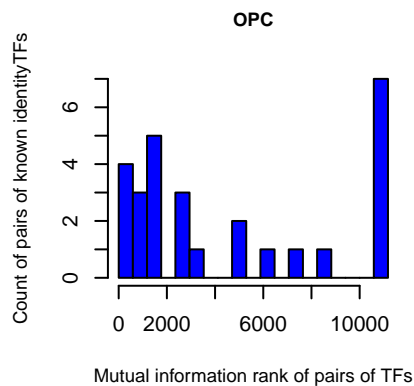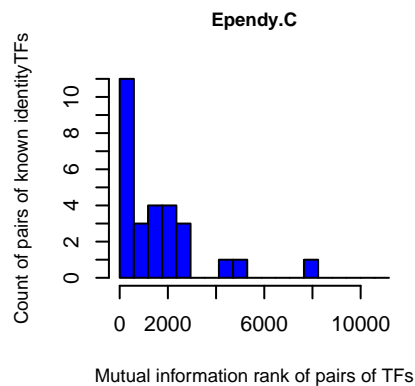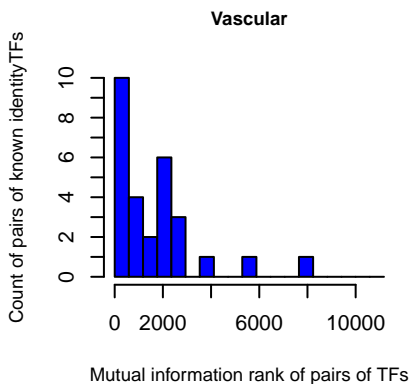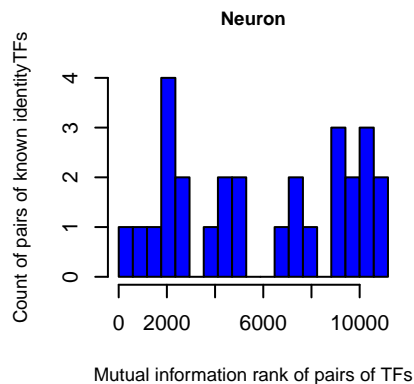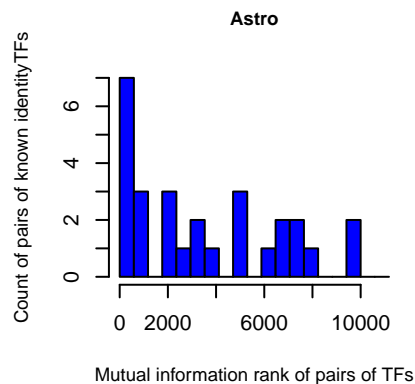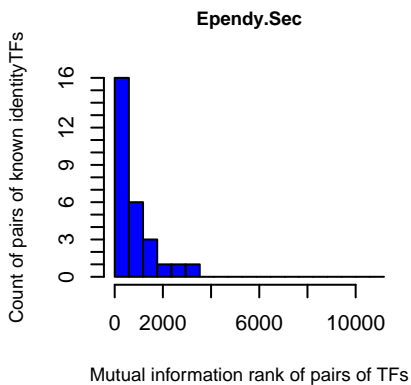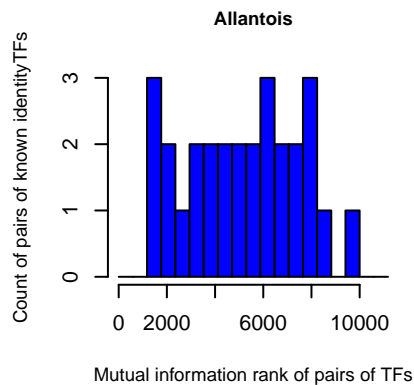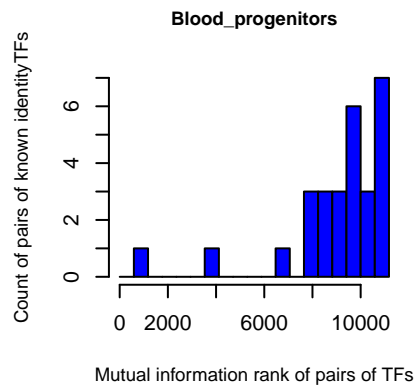

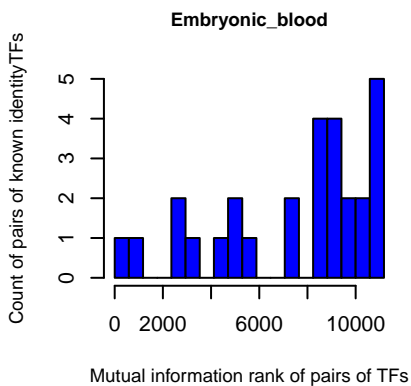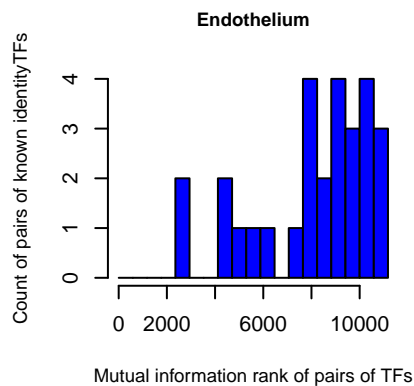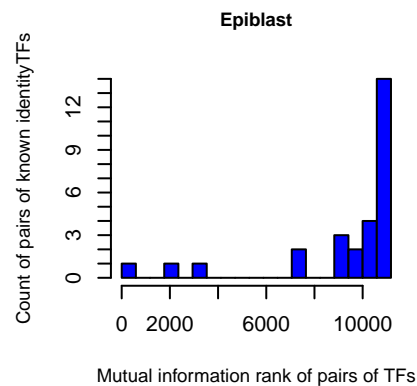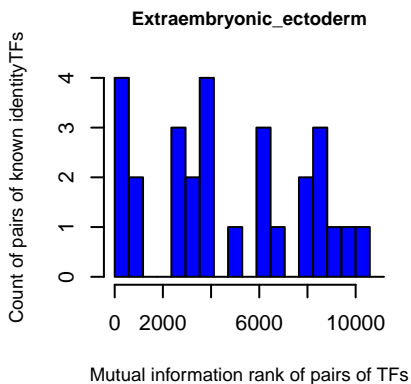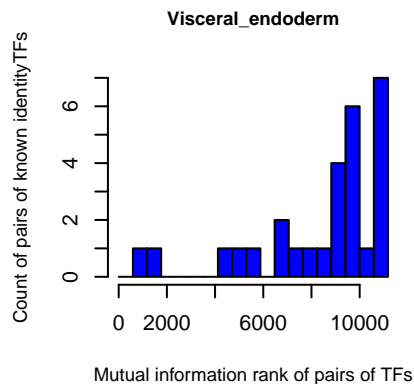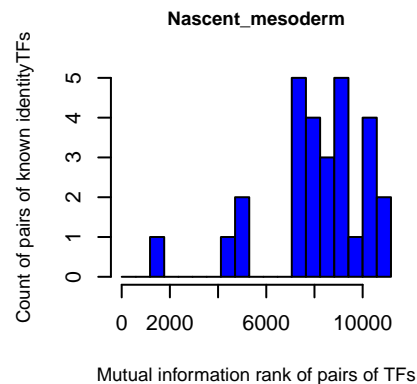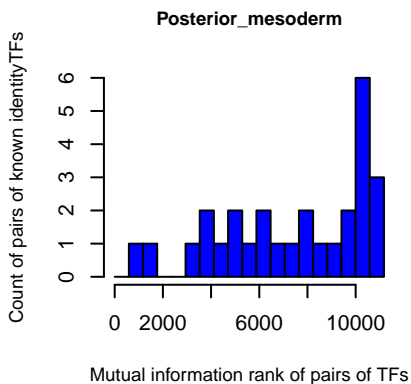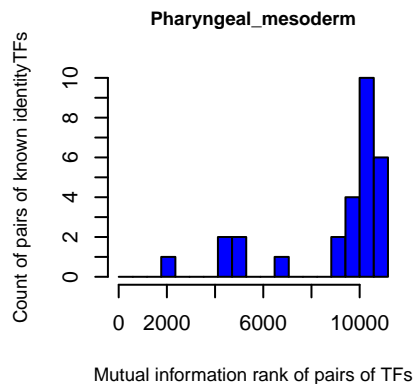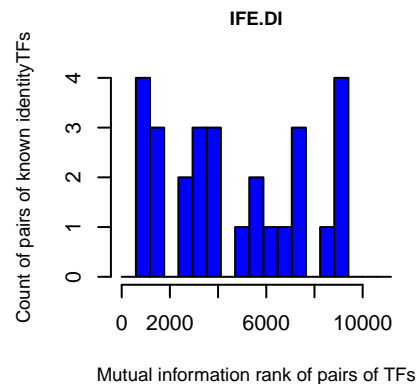

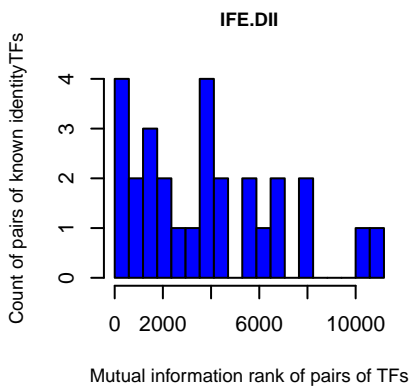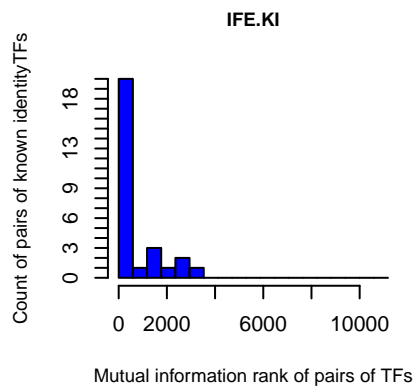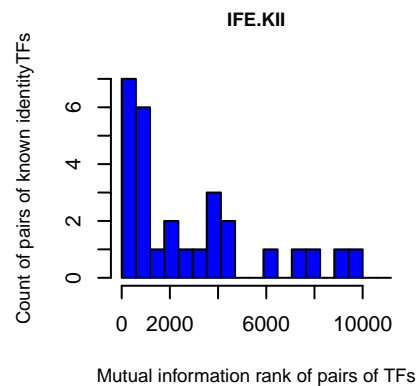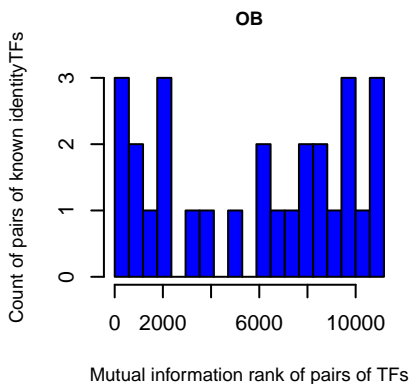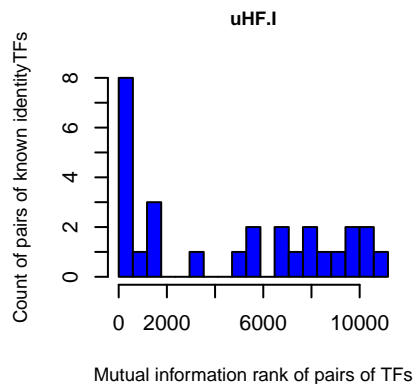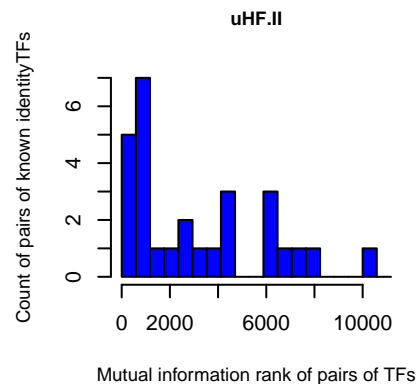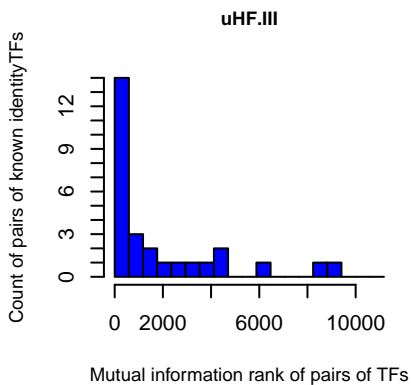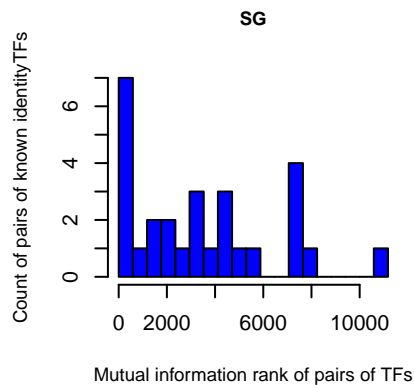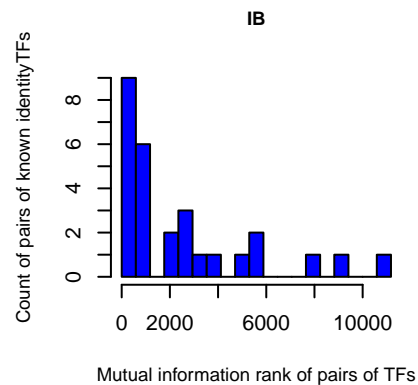

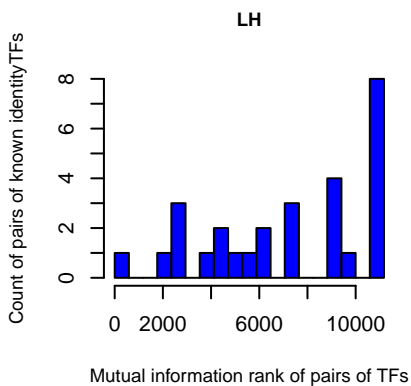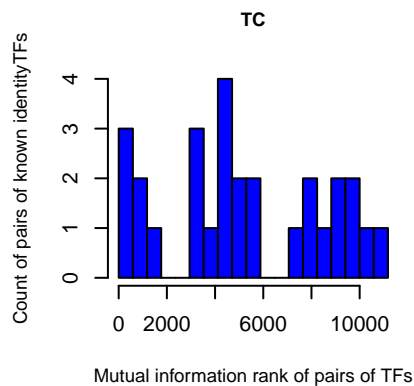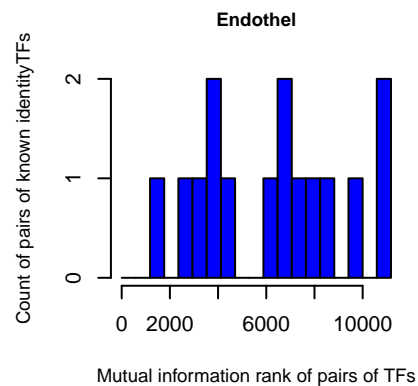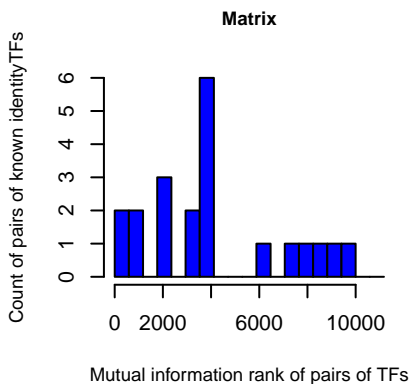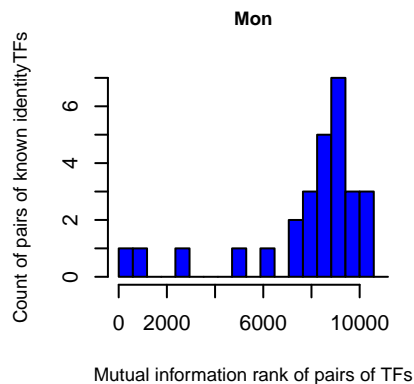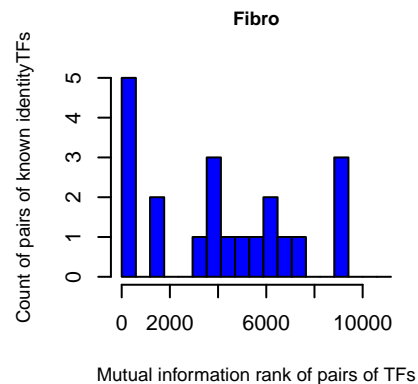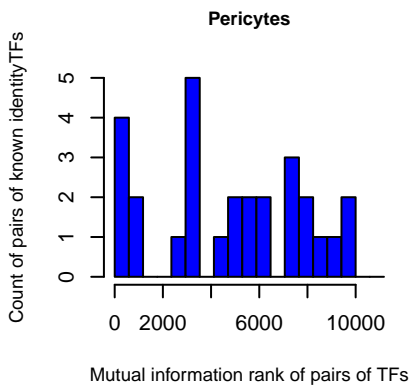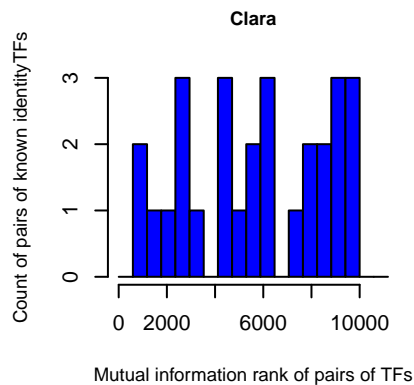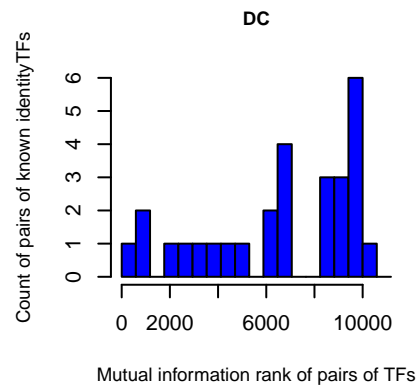

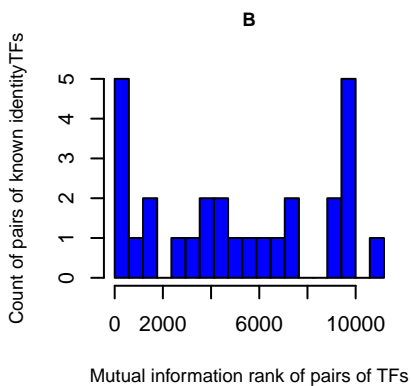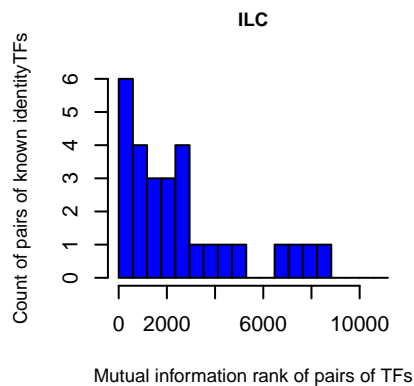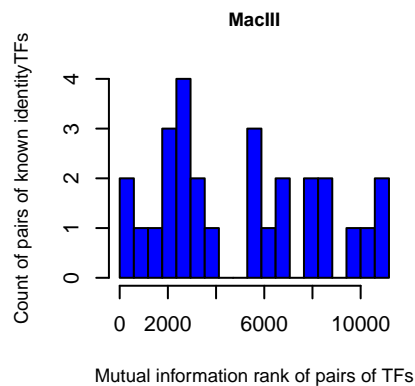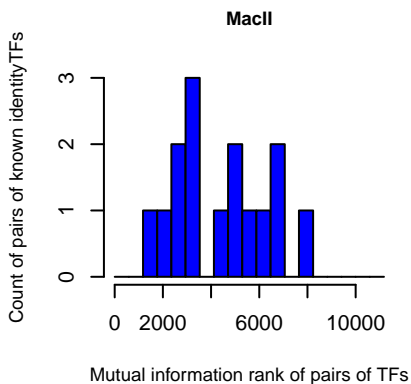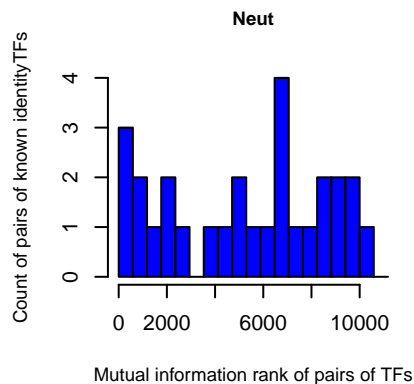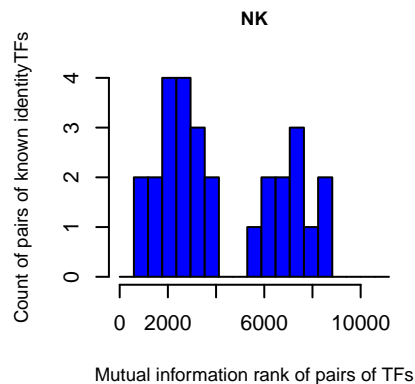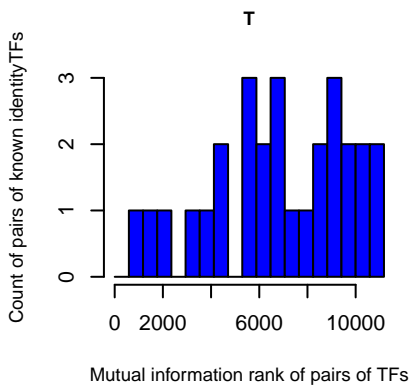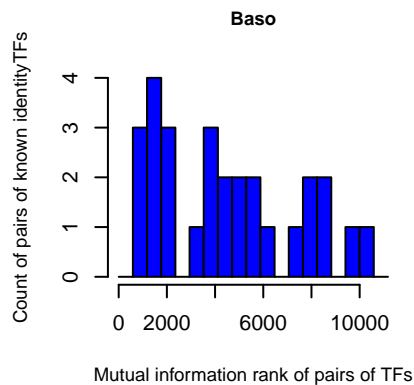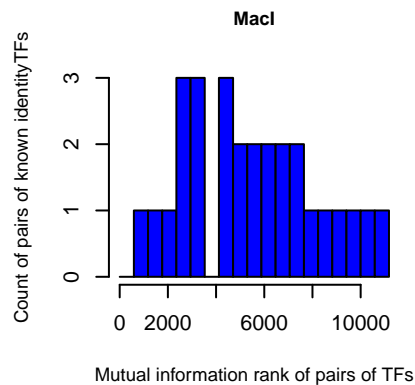

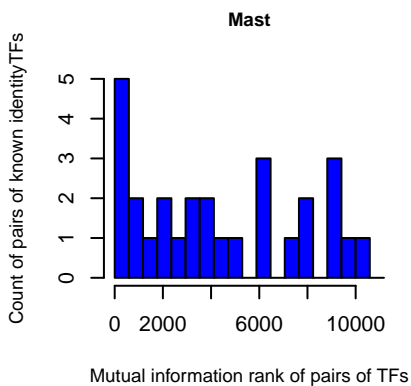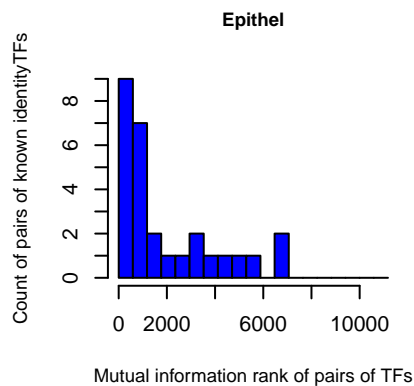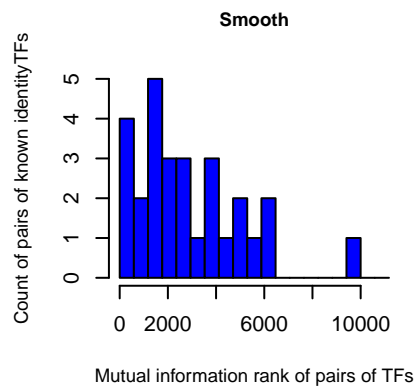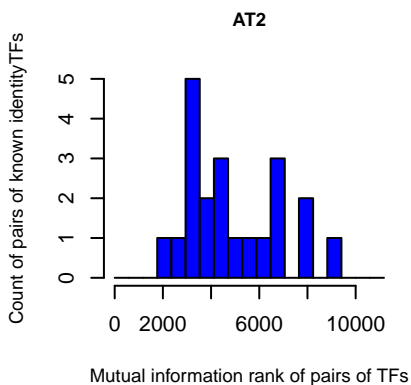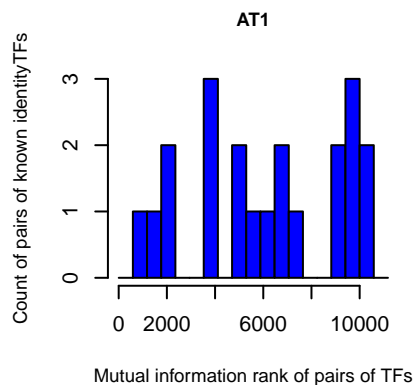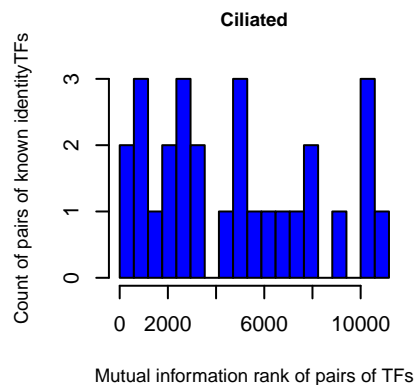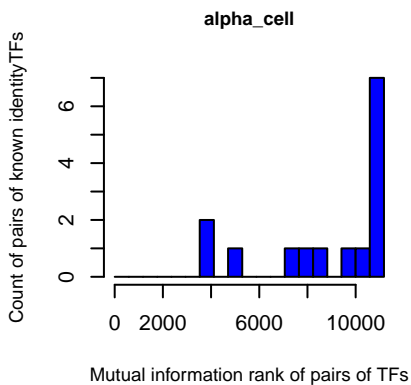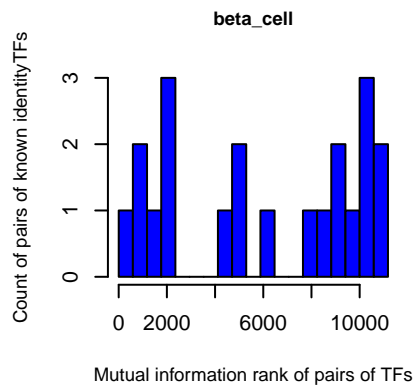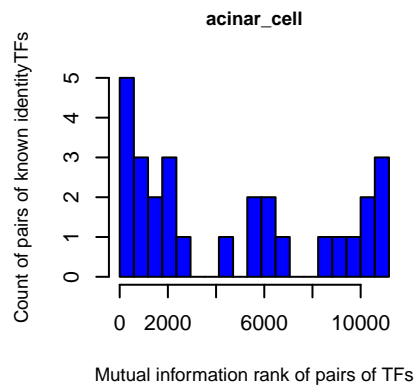

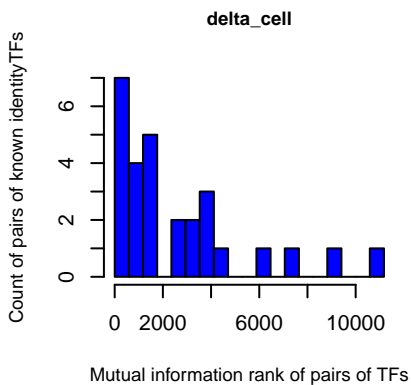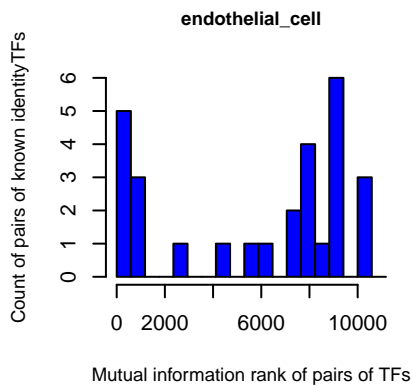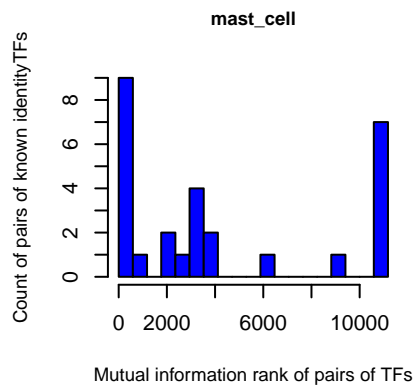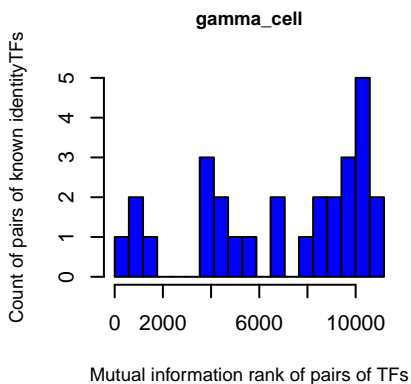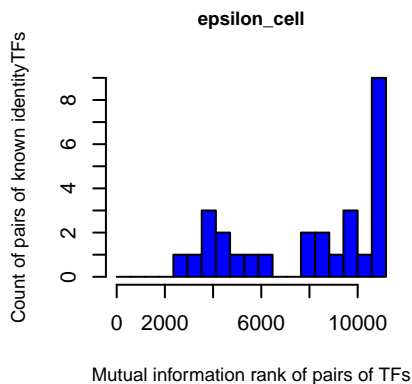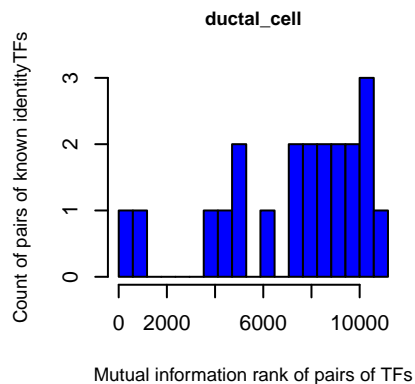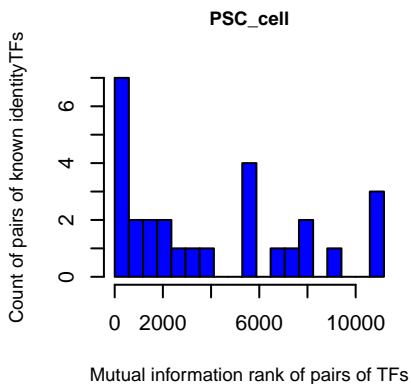

Supplement: Supplementary Data [file gkz147_supplemental_files.zip › Fig_S2.pdf]

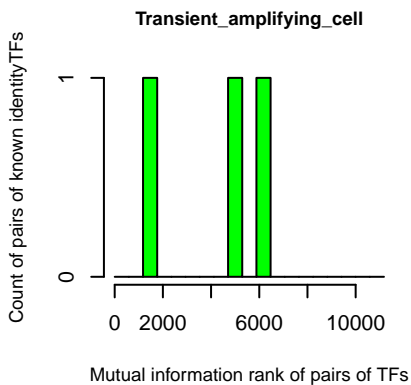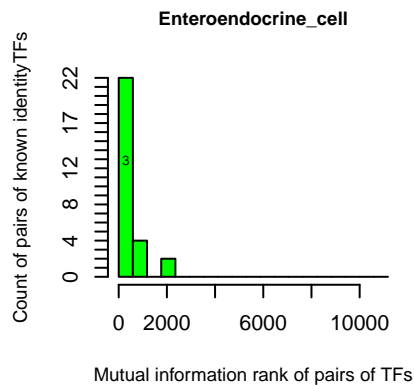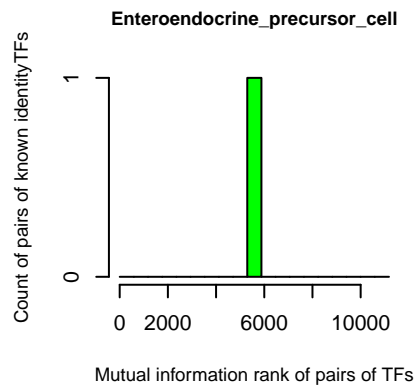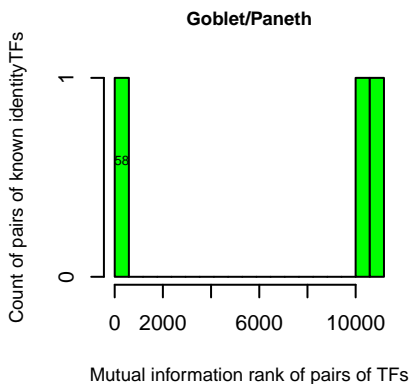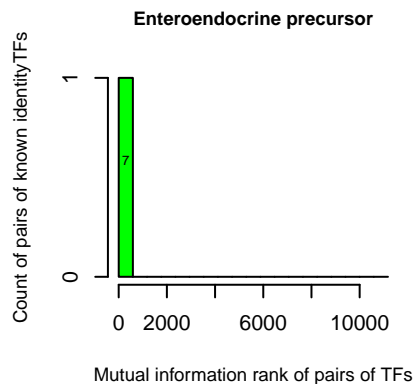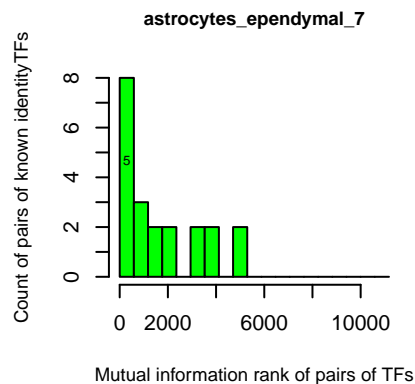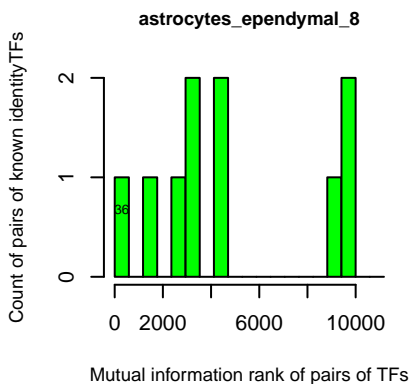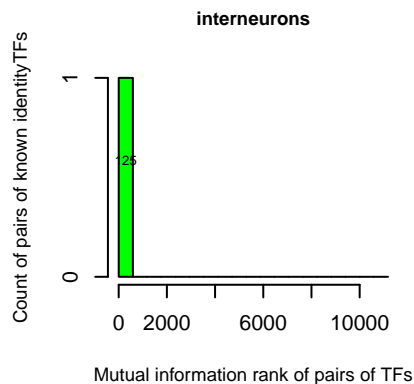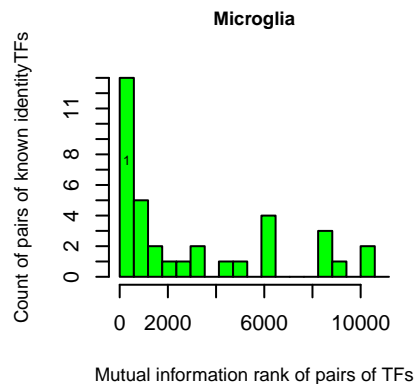

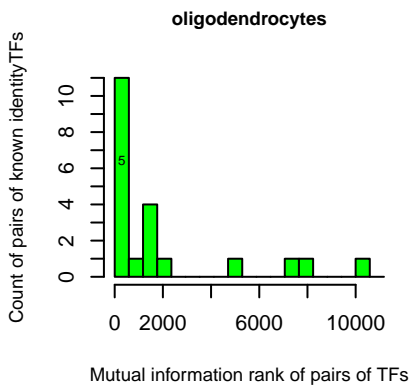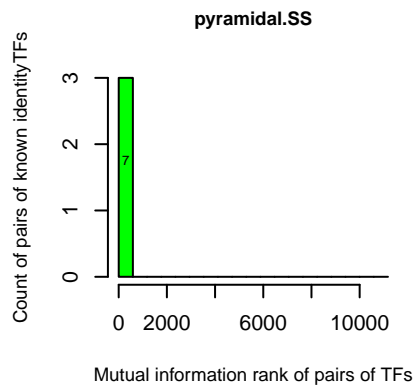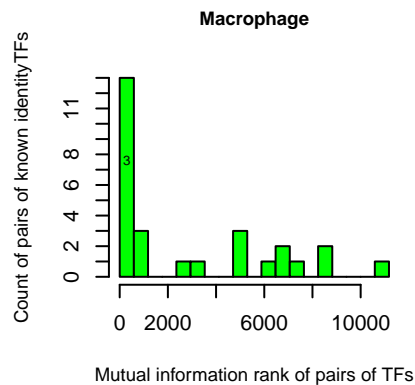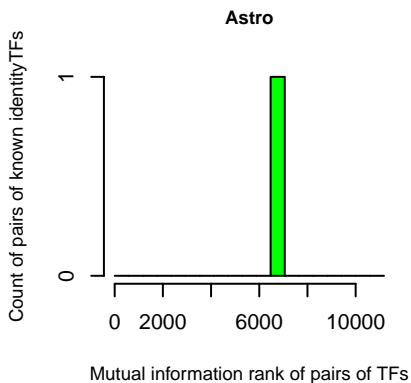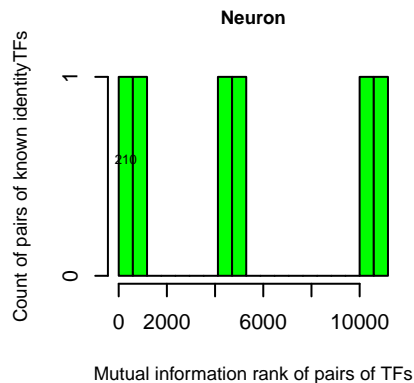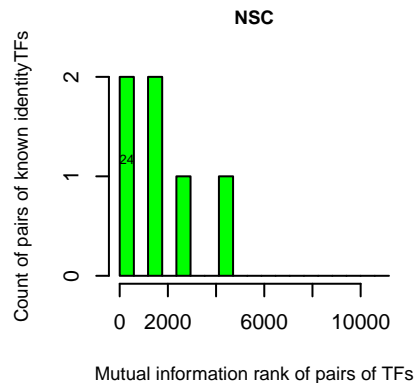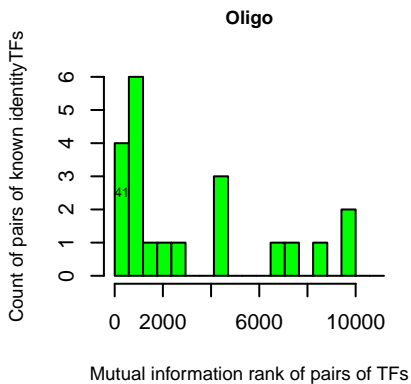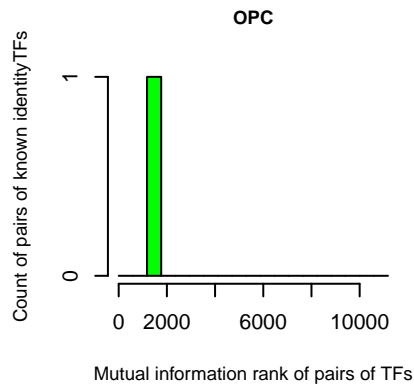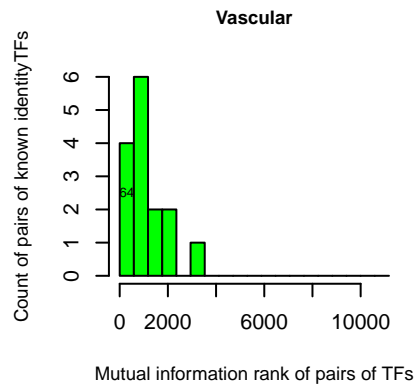

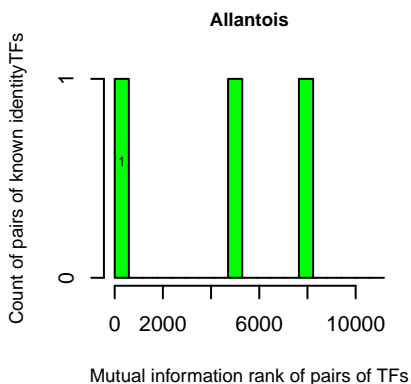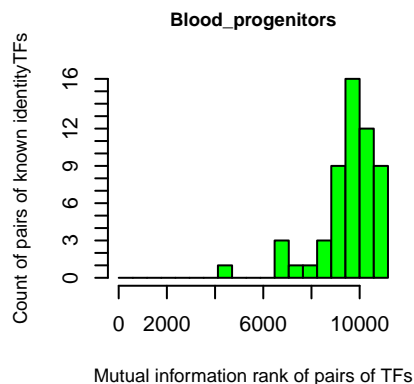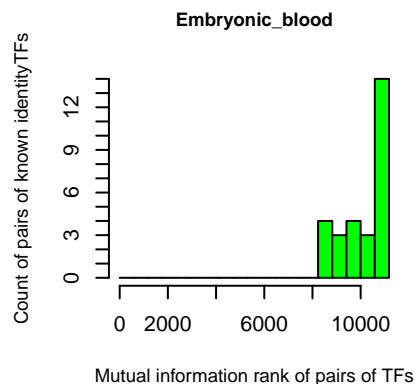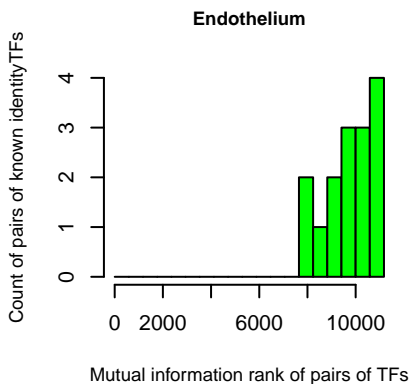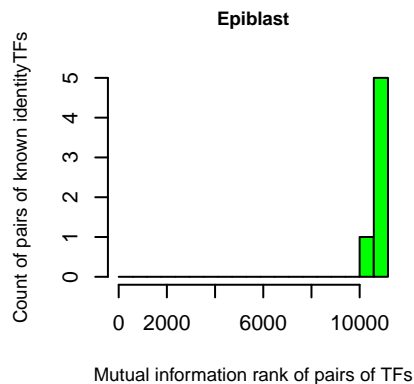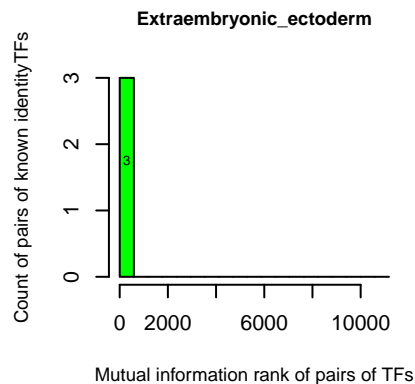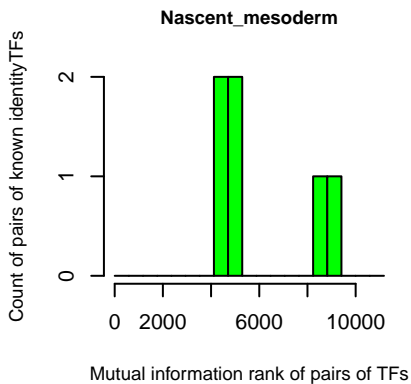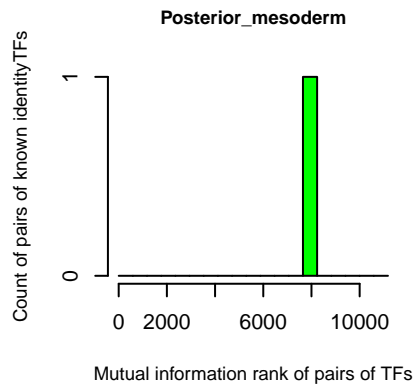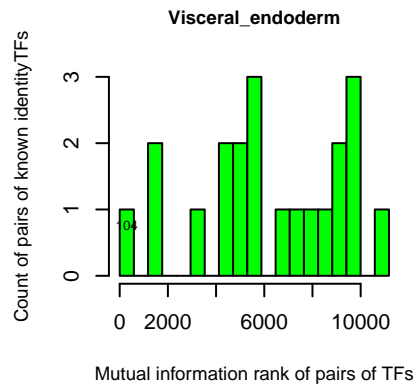

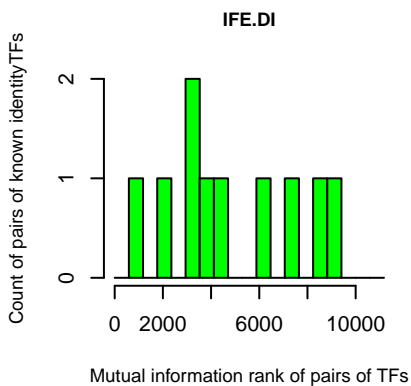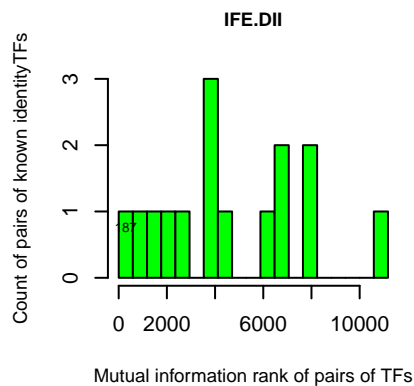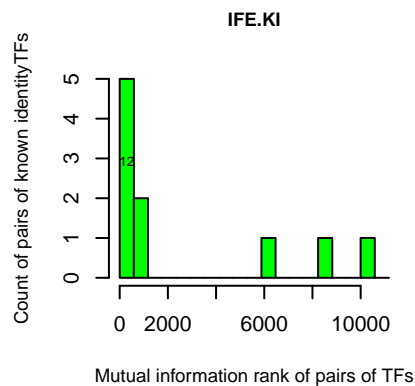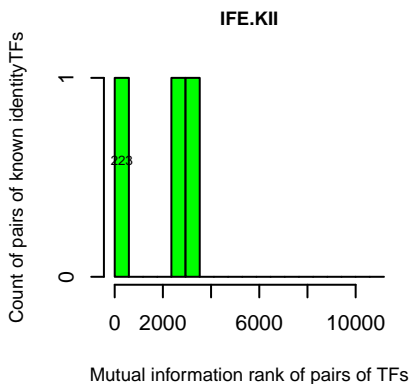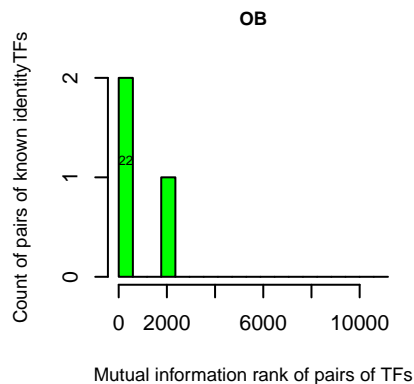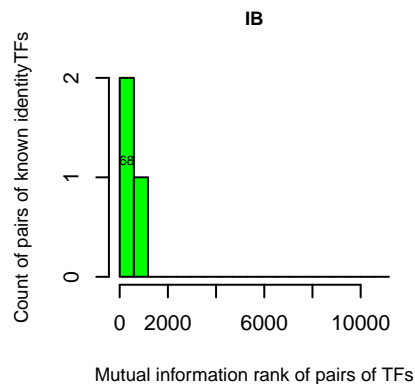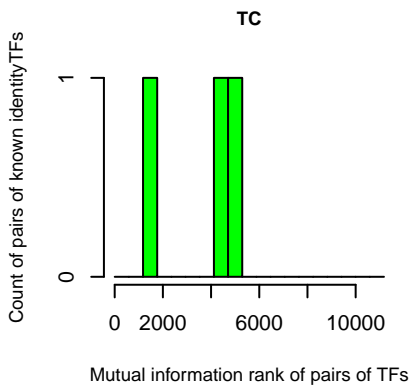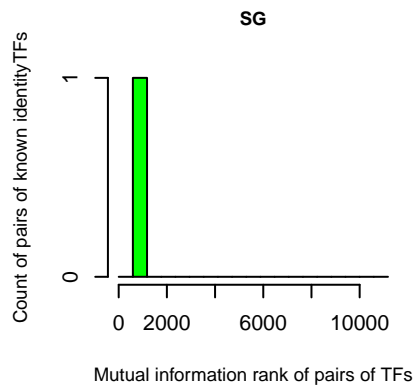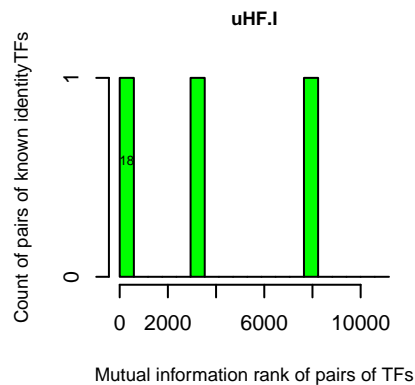

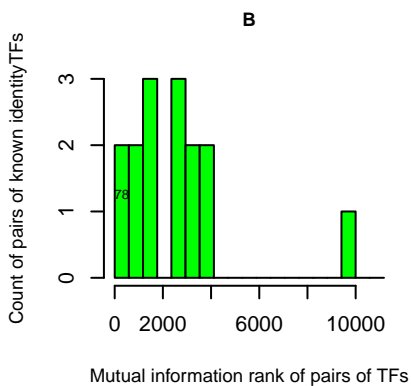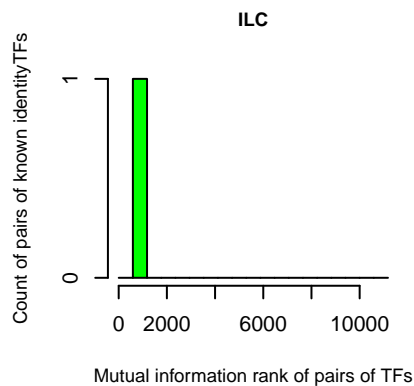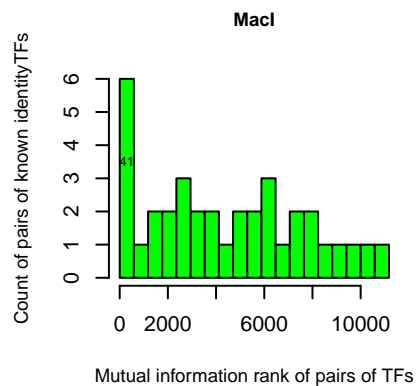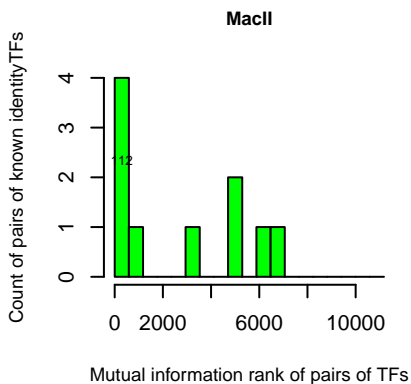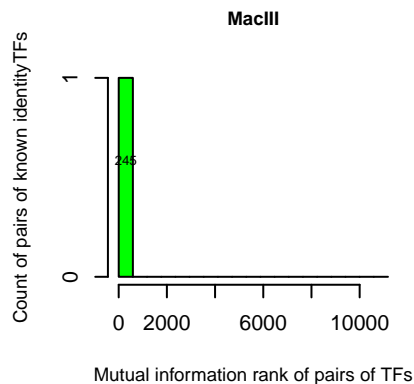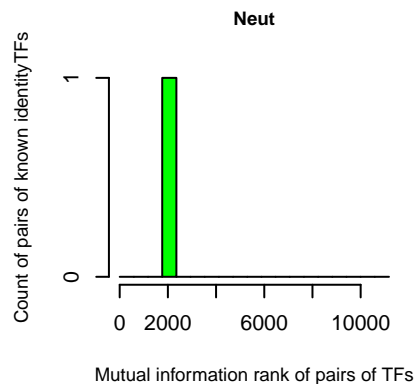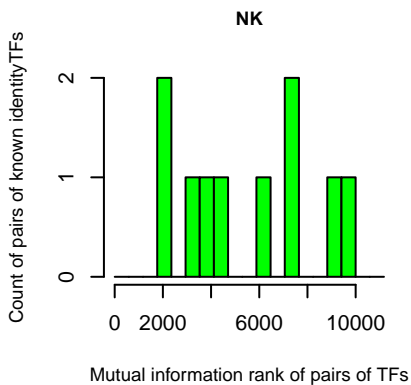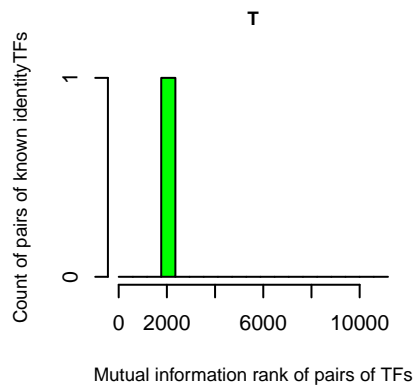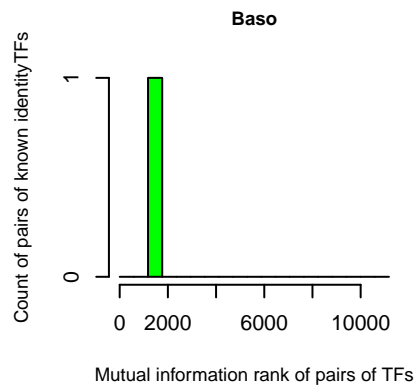

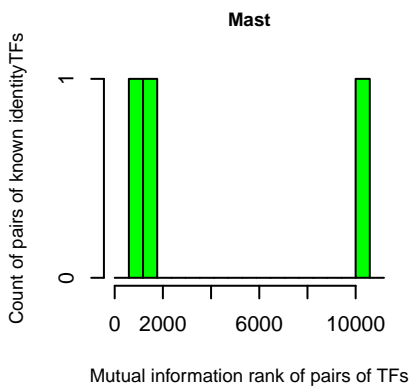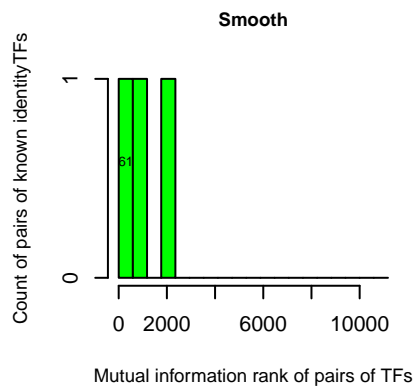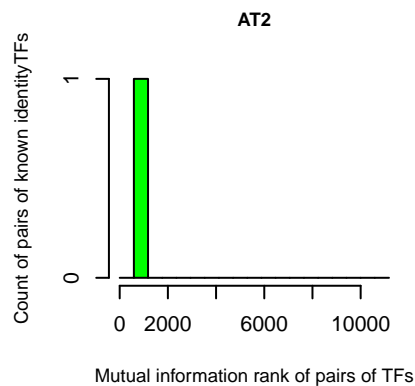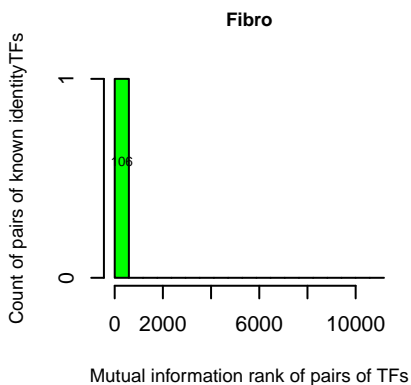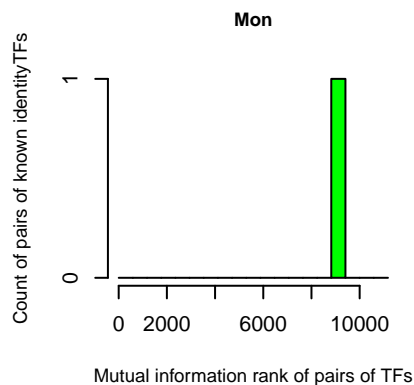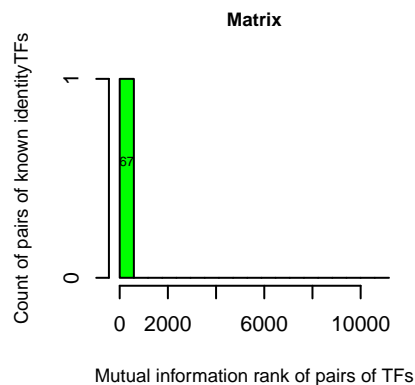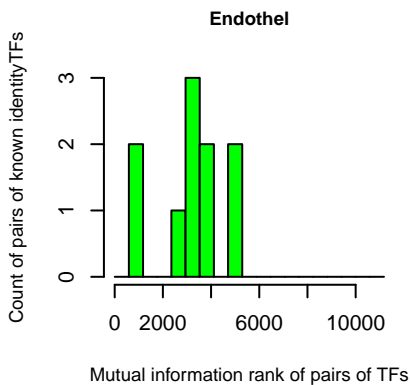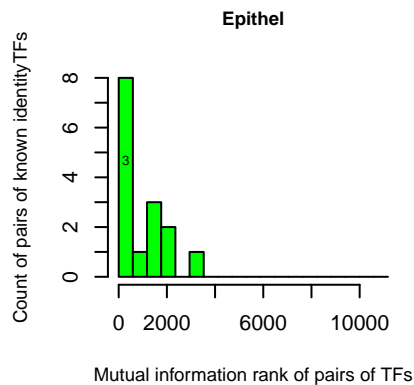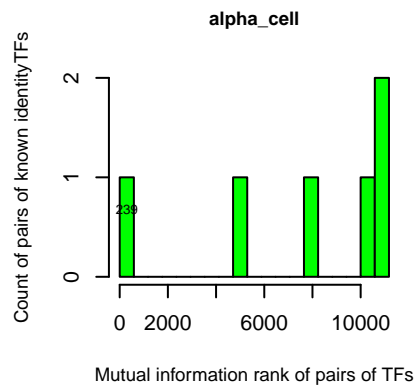

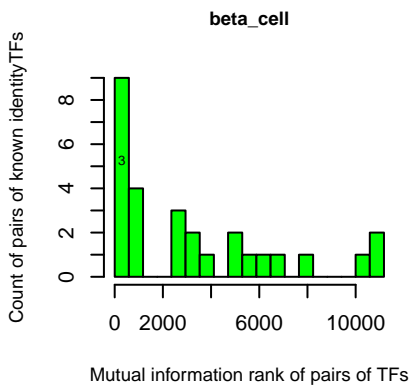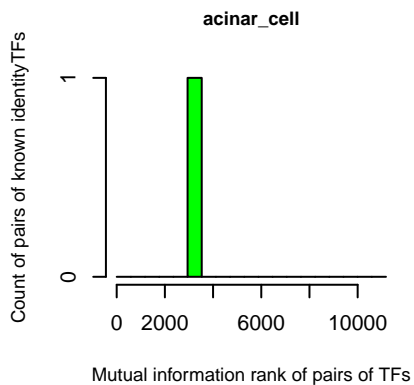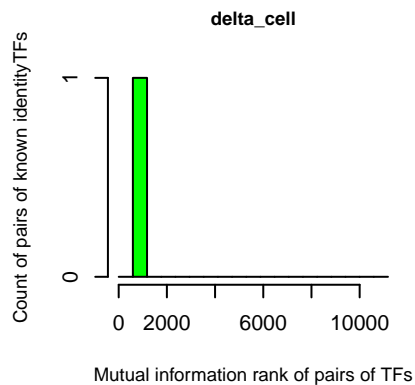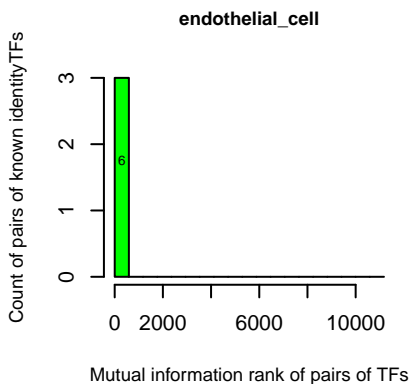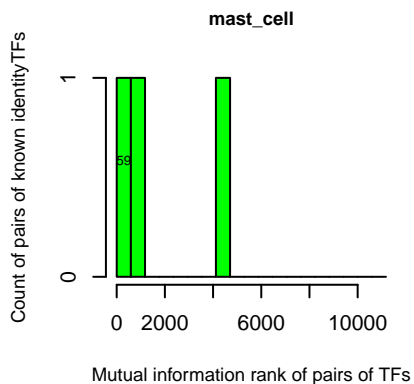

Supplement: Supplementary Data [file gkz147_supplemental_files.zip › Fig_S3.pdf]
